# Host-Specific Adaptation of *Legionella pneumophila* to Single and Multiple Hosts

**DOI:** 10.1093/molbev/msaf161

**Published:** 2025-07-03

**Authors:** Anaísa B Moreno, Kiran Paranjape, Martina Cederblom, Elisabeth Kay, Christian Dobre-Lereanu, Dan I Andersson, Lionel Guy

**Affiliations:** Department of Medical Biochemistry and Microbiology, Science for Life Laboratories, Uppsala University, Uppsala, Sweden; Department of Medical Biochemistry and Microbiology, Science for Life Laboratories, Uppsala University, Uppsala, Sweden; Department of Medical Biochemistry and Microbiology, Science for Life Laboratories, Uppsala University, Uppsala, Sweden; Centre International de Recherche en Infectiologie, Legionella Pathogenesis, Lyon, France; Department of Medical Biochemistry and Microbiology, Science for Life Laboratories, Uppsala University, Uppsala, Sweden; Department of Medical Biochemistry and Microbiology, Science for Life Laboratories, Uppsala University, Uppsala, Sweden; Department of Medical Biochemistry and Microbiology, Science for Life Laboratories, Uppsala University, Uppsala, Sweden

**Keywords:** host adaptation, *Legionella pneumophila*, experimental evolution, host–parasite relationships, host specificity

## Abstract

*Legionella pneumophila* is an endosymbiotic bacterial species able to infect and reproduce in various protist and human hosts. Upon entry into human lungs, they may infect lung macrophages, causing Legionnaires' disease (LD), an atypical pneumonia, using similar mechanisms as in their protozoan hosts, despite the 2 hosts being separated by a billion years of evolution. In this study, we used experimental evolution to identify genes conferring host specificity to *L. pneumophila*. To this end, we passaged *L. pneumophila* in 2 different hosts—*Acanthamoeba castellanii* and the human macrophage-like cells U937—separately and by switching between the hosts twice a week for a year. In total, we identified 1,518 mutations present in at least 5% of the population at the time of sampling. Forty-nine mutations were fixed in the 18 populations at the end of the experiment. Two interesting groups of mutations included (i) mutations in 4 different strain-specific genes involved in lipopolysaccharide (LPS) synthesis, found only in the lineages passaged with *A. castellanii* and (ii) mutations in the gene coding for LerC, a key regulator of protein effector expression, which was independently mutated in 6 lineages grown in presence of the macrophage cells. We propose that the mutations degrading the function of the regulator LerC improve the fitness of *L. pneumophila* in human-derived cells and that modifications in the LPS are beneficial for growth in *A. castellanii*. This study is a first step in further investigating determinants of host specificity in *L. pneumophila.*

## Introduction

To date, 65 species of *Legionella* have been discovered, half of which can cause disease in humans. Globally, 85% of cases of legionellosis are caused by *Legionella pneumophila*—with the exception of Australia and New Zealand where 30% of cases are caused by *Legionella longbeachae* (Fields et al. 2002; Den Boer and Yzerman 2004). *Legionella* causes 2 forms of legionellosis in humans—Legionnaires' disease (LD) and Pontiac fever. LD is presented as a severe pneumonia, with mortality of up to 15%, while Pontiac fever induces a milder flu-like illness. The worldwide occurrence of LD is difficult to calculate due to misdiagnosis and underreporting, but it has been established as a major contributor to both community- and hospital-acquired pneumonia (Falcó et al. 1991; Diederen 2008; Lau and Ashbolt 2009).

*Legionella* has a long history of adaptation to eukaryotes. The order encompassing *Legionella* and the related pathogen *Coxiella*, among others, is believed to have started colonizing eukaryotes almost 2 billion years ago (Hugoson et al. 2022). The ability of *Legionella* to infect humans has been associated with its long history of coevolution with protozoans. *Legionella* is able to control its host's cellular processes, such as vesicle trafficking. However, beyond the processes that are shared and highly conserved among all eukaryotes, *Legionella* can also control others that are absent in the protists it frequently encounters (Park et al. 2020). It is unlikely that *Legionella* has evolved these abilities simply from accidental transmission to humans and man-made aquatic environments in the past century, especially because humans are presumably evolutionary dead ends for *Legionella*, as it doesn’t spread from patient to patient. *Legionella* does not frequently meet the human host, and specific in-patient mutations occur despite the short infection cycle (Leenheer et al. 2023). This leads to a hypothesis that metazoan species are also natural hosts of *Legionella*, and these have been instrumental in furthering the evolution of *Legionella* to infect human macrophages (Mϕ) (Best and Abu Kwaik 2019).

The life cycle of *Legionella* replication is similar in all eukaryotic cells despite some host-specific differences. It is characterized by 2 distinct phases: replicative and transmissive (Oliva et al. 2018). Upon entry into the host cell, *Legionella* evades phagosome–lysosome fusion by forming a *Legionella*-containing vacuole (LCV). Shortly after the LCV is formed, it recruits host mitochondria and rough endoplasmic reticulum to surround its surface. In the LCV, *Legionella* undergoes exponential growth—the replicative stage. When the host's nutrients are exhausted, a new stage is triggered—the transmissive stage. There, *Legionella* is released from the LCV to the host's cytosol before exiting the cell using several mechanisms. In this transmissive state, *Legionella* is able to infect alveolar Mϕ and cause disease in humans. In laboratory conditions, *Legionella* also displays these replicative and transmissive characteristics during the exponential and stationary phases, respectively (Richards et al. 2013; Oliva et al. 2018).

The capacity of *Legionella* to evade lysosomes and modulate cellular processes in the host is conferred by the Dot/Icm system (Isberg et al. 2009). The latter is classified as a type IVB secretion system (T4BSS) and has 2 groups of loci—the *dot* (defect in organelle trafficking) and the *icm* (intracellular multiplication). In *Legionella*, this secretion system injects over 300 effector proteins into the host cell. These effectors play a role in the entry of *Legionella* into the host cell, formation of LCV, and exit from the host (Ensminger 2016). They also manipulate various cellular processes such as vesicle trafficking, protein translation, and ubiquitination pathways. About 10% of the coding capacity of *L. pneumophila* is used to express these effector proteins (Richards et al. 2013).

A large number of effector proteins are functionally redundant. This has been illustrated by experiments where deleting 31% of the effectors did not significantly impair the intracellular replication of *Legionella* in mouse Mϕ (O’Connor et al. 2011). Many effector proteins contain eukaryotic domains which are thought to facilitate modulation of host cells (Hubber and Roy 2010). These domains can combine to make new effectors, which could be one of the reasons why *Legionella* can infect such a wide range of hosts (Gomez-Valero et al. 2019).

An important part of *Legionella*'s infection cycle is the coordination and regulation of effector expression where 4 two-component systems (TCS) are central (Segal 2014). Together, they regulate the expression of over 100 genes (Feldheim et al. 2018) by sensing environmental stimuli and transducing the signal into the cell, often via an autophosphorylation mechanism. By timing the expression of the right effectors at the right time, they probably play an important role in host specificity.

The broad host range of *Legionella* is thought to have arisen from its lifestyle of host cycling, which selects against mutations that may decrease its fitness in the many protozoans it naturally infects. This was tested in an experimental evolution setup, where *Legionella* was passaged in mouse Mϕ for a year. The results showed rapid parallel evolution of *Legionella* and adaptive mutations that resulted in improved replication in Mϕ when compared to ancestral strains (Ensminger et al. 2012). Another evolution experiment involving *Legionella* and amoebae is the spontaneous endosymbiosis discovered by Jeon and Lorch (1967). Following an unexpected infection of one of their cultures of *Amoeba proteus* by an unknown bacterium (X-bacterium, now renamed ‘*Candidatus* Legionella jeonii’), a few amoebae survived but kept the bacterium, which turned out to be essential for the amoeba upon subsequent cultivation.

In this study, we aimed to explore the selective pressures imposed on the multihost endosymbiont *L. pneumophila*. By using experimental evolution, we sought to identify genetic determinants of host specificity in this bacterium. We cycled *L. pneumophila* in 2 different hosts separately and by repeated host switching. After 1 year of biweekly passaging, we sequenced the evolved lineages at the population level and tested the fitness effects of the most frequent genotypes.

## Results

### Distribution of Mutations

The starting population for all lineages consisted of a 1:1 mix of SYFP2- and dTomato-tagged *L. pneumophila* Paris strains. Each of the 6 lineages for all 3 hosts was passaged in their hosts for up to 1 year and sequenced at the population level at 2 or 3 time points, after ca. 10, 45, and 75 passages (supplementary table S1, Supplementary material online, supplementary fig. S1, Supplementary material online). All lineages passaged with human macrophage-derived cells (U937 cells, abbreviated as Mϕ) were sequenced at all 3 time points, while for the other 2 conditions, passaged with *Acanthamoeba castellanii* (*Ac*) and switching between the 2 hosts (alternation, Alt), only 1 population was sequenced at one of the 2 later time points.

We identified a total of 1,518 mutations that exceeded a 5% frequency in any population after removing low-frequency mutations (<20%) in 1 sample (Alt_D, t1), which was contaminated with another strain of *L. pneumophila*. The number of mutations reaching 5% frequency ranged from 4 to 102 per population and time point, with a median of 28.5 (supplementary fig. S2, Supplementary material online). The number of mutations appeared more variable and on average slightly higher at time point 1 than 3 (supplementary fig. S3a, Supplementary material online). The fraction of nonfixed mutations is also higher at time point 1 than 3 (supplementary fig. S3b, Supplementary material online). Small indels accounted for only 4% of the mutations (*n* = 62). As expected, among SNPs, transitions (987, 68%) were more frequent than transversions (468, 32%). The number of AT → GC mutations (*N* = 599, 50.4%) was very similar to the reverse (GC → AT; *N* = 589, 49.6%). The numbers were also very similar when excluding repeated regions and potential recombination hotspots prone to recombination (GC → AT: 270, 50.6%; AT → GC: 264, 49.4%). Very few mutations (15) were found on the *L. pneumophila* Paris pLPP plasmid. All of them appeared with frequencies <15%. All further analyses focused on the chromosomal mutations. When mapping reads from the fluorescent-tagged mutants (ancestors, see supplementary fig. S1, Supplementary material online) to the reference genome, a few SNPs were detected: 15 in the SYFP2 clone and 7 in the dTomato one, with low frequencies (on average 9.8% and 7.5%, respectively). The median sequencing coverage was 64×, with only 2 lineages (Mϕ_A, t1 and Mϕ_C, t1) below 30×.

### Mutational Hotspots

Mutations were not uniformly distributed along the chromosome, and many clustered around several hotspots (supplementary fig. S4, Supplementary material online). In total, mutations in these hotspots accounted for 751 or 49.4% of all mutations, but notably, none were fixed. Eleven hotspots corresponded to intergenic regions upstream or downstream of the GIY-YIG nuclease family protein, a homing endonuclease/mobile element (Dunin-Horkawicz et al. 2006). In total, 97 mutations occurred in these 11 hotspots. A second mutation hotspot was a gene corresponding to locus tag lpp1100, coding for an ankyrin repeat-containing protein, which displayed 142 mutations. The ankyrin repeats (19 repeats of 105 nucleotides; supplementary fig. S5, Supplementary material online) are presumably different enough so that the variants called by breseq represent true mutations and not mapping errors. The third hotspot for mutations was one of the 5 tetratricopeptide repeat (TPR; 19 mutations) proteins in *L. pneumophila* str. Paris, lpp2912. The fourth hotspot was a repeated protein containing a domain of unknown function (DUF1566; 36 mutations) widely present in *Gammaproteobacteria*. Finally, the fifth group of hotspots was constituted of 3 occurrences of a larger segment containing a transposase and a type II-like restriction endonuclease (DUF559; 457 mutations).

To further study whether these repeated regions may be recombination hotspots, leading to chromosomal rearrangements, we analyzed the junctions predicted by breseq (supplementary fig. S6, Supplementary material online). Briefly, breseq predicts recombinations or junctions by identifying reads with split-read alignments, where one end of the read maps to one part of the reference and the other end to another (Deatherage et al. 2015). Junctions mapping to regions of the chromosome within 5 kb were discarded to focus on long-range recombinations. We identified 61 junctions in 20 out of the 37 populations for which we have sequence data. Of these, 27 (44%) had both ends in the vicinity (2 kb) of one of the potential recombination hotspots, 13 (21%) had one end close to one of the hotspots, while the rest (21, 34%) linked other parts of the chromosome. To control for false positives due to the repetitive nature of these regions, we performed the same analysis on 2 sets of reads obtained from single colonies of the 2 ancestral strains carrying the fluorescent markers. Breseq did not detect any junctions in any of these 2 datasets. The average read coverage in these samples was comparable to the read coverage obtained from population sequencing of the lineages (average coverage for the dTomato, 35×; for SYFP2, 90×; population sequencing, average of 70×; range 20× to 120×).

### Frequent and Fixed Mutations

The frequency of variants varied greatly, but 20, 35, and 36 mutations appeared fixed after t1, t2, and t3, respectively; this corresponded to 1.2, 2.7, and 2.8 mutations per population. After the last time point (t3 for *Ac* and Mϕ and t2 for most Alt populations), 10, 22, and 15 mutations were fixed in *Ac*, Mϕ, and Alt conditions, respectively. In some populations (e.g. Mϕ_A and Alt_D at t1 and *Ac*_D and Mϕ_C at t3; supplementary fig. S3b, Supplementary material online), groups of variants were found at a higher frequency but were not fixed, suggesting 2 subpopulations with distinct mutations coexisting in the same culture.

**Fig. 1.**
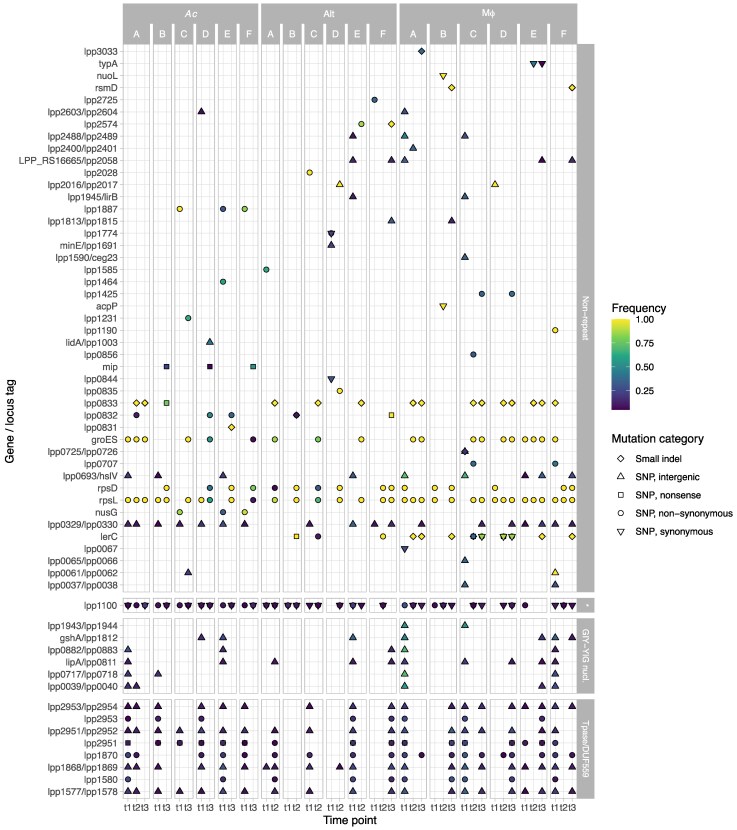
Frequency of mutations appearing at least once within a gene or an intergenic space, with a frequency in the population of at least 25%. Mutations occurring within a gene are labeled with the locus tag or the gene name (if available). Mutations occurring in an intergenic space are labeled with the 2 bordering gene names or locus tags. If a gene/intergenic space is mutated several times, with different types of mutations (e.g. *lerC*), the symbols are stapled on each other. The shape of the points corresponds to the type of mutation. The color of each point corresponds to the frequency of the mutation. Yellow shapes are fixed mutations. Mutations occurring in nonrepetitive regions are shown at the top and repetitive regions at the bottom. The repetitive region marked with a “*” corresponds to lpp1100. The time point t1 corresponds to 128, 124, and 113 generations for *Ac*, Alt, and Mϕ, respectively; t2 corresponds to 517, 481, and 270 generations for *Ac*, Alt, and Mϕ, respectively; t3 corresponds to 798, 799, and 444 generations for *Ac*, Alt, and Mϕ, respectively (supplementary table S2, Supplementary material online).

In addition to the potential recombination hotspot regions mentioned above, other genes were also mutated several times with higher frequencies (Fig. 1). Most fixed mutations (92.3%) were small indels or nonsynonymous mutations, while intergenic or synonymous mutations were more common at lower frequencies (Table 1), as expected if genetic drift was not prevalent. Remarkably, synonymous mutations dropped from 20.1% among all mutations to only 2.2% (*n* = 2) among fixed mutations. Many nonsynonymous mutations occurred independently in the same gene (e.g. *rpsL*, *lerC*, and lpp0832) (Fig. 1).

**Table 1:** Number and frequency of mutations per category

Mutation category	*N* (>5%)	%	*N* (>25%)	%	*N* (fixed)	%
Small indel	62	4.1	29	3.7	23	25.3
SNP intergenic	611	40.4	404	52.1	3	3.3
SNP nonsense	42	2.8	21	2.7	2	2.2
SNP nonsynonymous	492	32.6	240	30.9	61	67.0
SNP synonymous	304	20.1	82	10.6	2	2.2
*Total*	1,511	100.0	776	100.0	91	100.0

Mutations were counted for all 3 time points. The 3 pairs of columns represent the number and percentage of mutations with frequency over 5%, with frequency over 25%, and fixed in the population (100%), respectively.

In total, 48 mutations (representing 13 distinct mutations in 11 different genes) were fixed in the population after the last time point (Table 2). This corresponds to about a year of passaging in the 3 conditions: ∼450 generations in Mϕ (t3), ∼480 generations in the switching condition (Alt, t2), and ∼800 generations in *Ac* (t3) (supplementary table S1, Supplementary material online).

**Table 2:** Fixed mutations

Gene	Product	Position^a^	Category^b^	Position^c^	*N* fixed^d^
*lerC*	Response regulator LerC	119,826	Small indel	61	4
		119,903	SNP nonsense	46	1
*rpsL*	30S ribosomal protein S12	451,837	SNP nonsyn	43	7
		451,972	SNP nonsyn	88	7
*rpsD*	30S ribosomal protein S4	467,982	SNP nonsyn	205	7
*groES*	Co-chaperone GroES	822,976	SNP nonsyn	45	7
lpp0831	Hypothetical protein	930,446	Small indel	808	1
lpp0832	Phosphotransferase	931,503	SNP nonsense	203	1
lpp0833	N-acetylneuraminate synthase family protein	933,165	Small indel	182	8
lpp0835	Hypothetical protein	935,076	SNP nonsyn	228	1
lpp2016/lpp2017	Hypothetical protein/cation diffusion facilitator family transporter	2,283,953	SNP intergenic	162	1
lpp2574	HAMP domain-containing histidine kinase	2,928,346	Small indel	594	1
*rsmD*	16S rRNA (guanine(966)-N(2))-methyltransferase RsmD	3,107,822	Small indel	42	2

Mutations shown are those fixed at the last time point for each lineage. Genes lpp0831, lpp0832, lpp0833, and lpp0835 are part of the same LPS biosynthesis operon. ^a^Position along the *L. pneumophila* Paris chromosome (NC_006368). ^b^SNP, single-nucleotide polymorphism; (non-)syn, (non-)synonymous. ^c^Position along the protein sequence or the nucleotide sequence (for indels and intergenic sequences). ^d^Number of fixed mutations across populations.

**Fig. 2.**
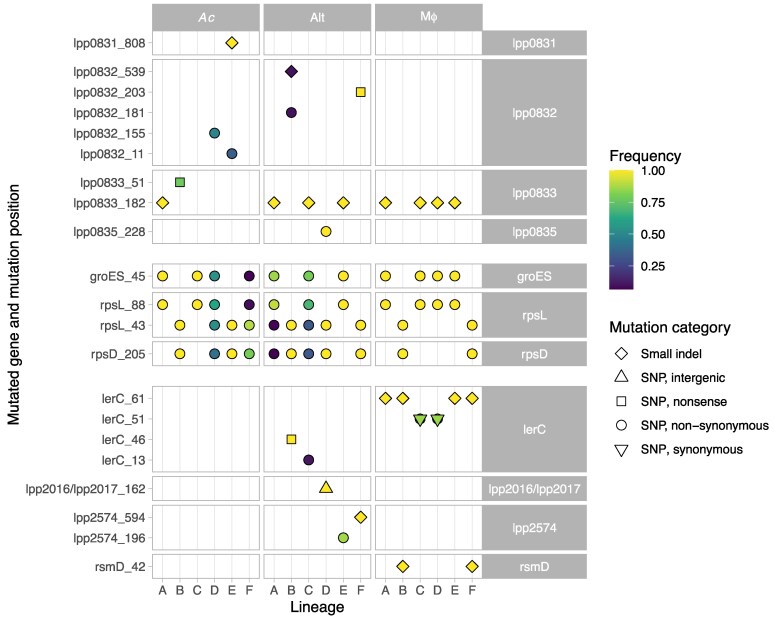
Genes with fixed mutations in the 3 conditions at the last sequenced time point. Mutations occurring within a gene are labeled with the locus tag or the gene name (if available) on the right side of the figure; mutations occurring in the same gene at different positions (*rpsL*, *lerC*) are shown on different lines, and the position of the mutation is shown on the left side of the panel. Mutations are grouped according to the condition in which they were found: in lineages passaged in *Ac* at the top, in lineages passaged in Mϕ at the bottom, and in both in the middle. For in-gene SNP mutations, the position of the amino acid whose codon has been mutated is indicated. For the intergenic mutation, the position is the number of nucleotides after the upstream gene; for the small indels, the position refers to the nucleotide position within the gene for the start of the indel. Mutations occurring in an intergenic space are labeled with the 2 bordering gene names or locus tags. The shape of the points corresponds to the type of mutation. The lerC_51 mutation is a double mutation in the same codon at the second and third codon positions, resulting in a nonsynonymous mutation. The color of each point corresponds to the frequency of the mutation. Yellow shapes are fixed mutations.

As expected, most fixed mutations were nonsynonymous or nonsense mutations or small intragenic indels (Fig. 2). Two synonymous mutations (in *acpP* and *nuolL*, Fig. 1) appeared to be fixed in the Mϕ_B population but were absent at the third time point. One intergenic mutation was found, 162 and 187 nucleotides upstream of 2 genes, lpp2016 and lpp2017, respectively (Fig. 2). According to in silico predictions, the latter gene is the first in a 2-gene operon (lpp2017 and lpp2018), both involved in ion transport (Price et al. 2005). The former gene, lpp2016, has no described function and appears to be strain-specific in *L. pneumophila*; it is present in subspecies *pascullei* and in strains Paris, Alcoy, and Corby but absent in Philadelphia (Altschul et al. 1997).

### Two Pairs of Mutations in Ribosomal Protein and Chaperonin Genes Are Mutually Exclusive

The distribution of mutations showed 2 distinct patterns of mutations, both of which were present under all 3 conditions (Fig. 2). In one (referred to as RpsL43/RpsD), the 30S ribosomal protein S12 RpsL was mutated at position 43, where the lysine is replaced by a threonine (K43T), while the 30S ribosomal protein S4 RpsD is mutated at position 205, where a serine replaces a tyrosine (S205Y). In the other pattern (RpsL88/GroES), RpsL has another mutation, where the lysine at position 88 is replaced by an arginine (K88R), while the co-chaperone GroES has a mutation at position 45 (A45T). Every population harbored one or the other pair of mutations, or a mix of both, at the last time point. The details of these mutations, their occurrence, and effects are presented and discussed in Supplementary material online.

### Mutations in the LPS Synthesis Gene Cluster

**Fig. 3.**
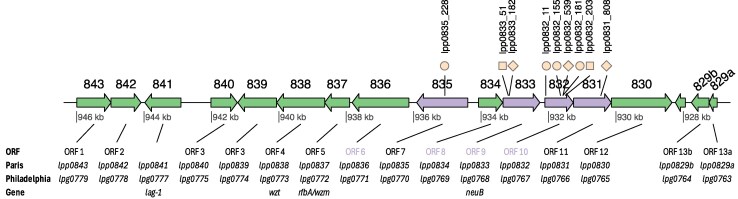
Genetic map of the variable part of the LPS biosynthesis cluster in *L. pneumophila* Paris. The lpp number is shown above the genes (843: lpp0843, etc.). Genes in purple harbored mutations in the evolution experiment conducted in this study. Location of the individual mutations (shown above the gene) is indicated with a black line. The symbols show the type of mutation: circle, nonsynonymous; square, nonsense; diamond, small indel. For each gene, the corresponding ORF number (as in e.g. Petzold et al. 2013), the lpp (Paris) and lpg (Philadelphia) numbers, as well as the gene name (if available), is shown under the map.

Four linked genes harbored fixed mutations: lpp0831 (a small indel), lpp0832 (1 fixed nonsense mutation and 4 nonfixed mutations: 1 small indel and 3 nonsynonymous mutations), lpp0833 (1 fixed small indel in many populations, as well as a nonfixed nonsense mutation in *Ac*_B), and lpp0835 (1 nonsynonymous mutation) (Figs. 2 and 3). These 4 genes belong to the variable segment of the lipopolysaccharide (LPS) synthesis gene cluster. The mutation in lpp0833 was fixed in 8 out of 18 lineages and only found in populations which harbored the RpsL88/GroES genotype. However, in 2 of these lineages (Alt_A and Alt_D), the lpp0833 mutation was found in 100% of the population, where the 2 genotypes RpsL88/GroES and RpsL43/RpsD coexisted. Thus, the lpp0833 was also found in the Rpsl43/RpsD background in these 2 lineages. The other mutations in the LPS synthesis operon were found exclusively in the lineages passaged in *Ac* or during switching conditions.

### Mutations in LerC, a Response Regulator of the Effector Regulatory Network

Four distinct mutations in LerC were found at the last time point: an A13D nonsynonymous change (lerC_13), a 13 nt insertion after nucleotide position 61 in the gene (codon position 21; lerC_61), a C46* nonsense mutation (lerC_46), and a double SNP, resulting in a nonsynonymous V51G mutation (lerC_51). All Mϕ lineages harbored one LerC mutation, which was fixed in 4 of them; 2 of the Alt lineages harbored one (B and C, fixed in B), but none was found in the *Ac* lineages (Figs. 1 and 2). All these mutations were absent at the first time point.

The 2 nonsynonymous lerC_13 and lerC_51 mutations seemed to have little effect on the structure of the LerC protein (supplementary fig. S7a and d, Supplementary Material online). The lerC_46 and lerC_61 mutations yielded a short peptide at the N-terminal (45 and 20 residues, respectively). However, longer proteins (88 and 122 residues, respectively) might possibly be translated from alternative start codons to the end of the protein. The resulting lerC_61 mutant protein would lack the first 2 β-strands and the first α-helix (supplementary fig. S7b, Supplementary material online), while the lerC_46 mutant protein would lack an additional α-helix and the conserved aspartic acid residue at position 53 (supplementary fig. S7d, Supplementary material online), which is essential for the phosphorylation and function of LerC (Feldheim et al. 2018).

### Other Mutations

**Fig. 4.**
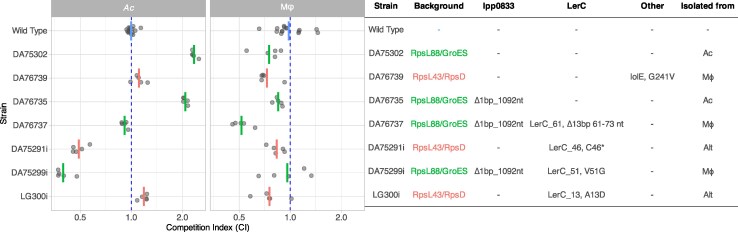
Competition index (CI) of different isolated strains evolved during the evolution experiment. Evolved strains of *L. pneumophila* Paris were competed in the Mϕ cells and *Ac* against their ancestral strain. Each replicate is represented by a grey dot, and the average is shown as a red or green bar, depending on the background. CI is calculated as the ratio of the fluorescence of the mutant (evolved) strain to the fluorescence of the wild type (ancestor) strain after 64 h of growth, divided by the same ratio at the beginning of the experiment. The blue dashed line is set at CI = 1, where evolved clones grow as much as the ancestors. A CI of 0.5 means that the evolved strain grew half as much as its ancestor. Mutations present in each strain are described in the table on the right panel.

Three other regions harbored fixed mutations at the last time point (Table 2; Fig. 2). The first one was an intergenic region upstream of both lpp2016 and lpp2017, where the mutation could play a role in regulating either or both genes. The second was the gene lpp2574, which encodes a HAMP domain-containing histidine kinase. Two different mutations were found in this gene: a 1 nt insertion in the middle of the gene (lpp2574_594), fixed in Alt_F, and a nonsynonymous R196S (lpp2574_196), present in 86% of the Alt_E population. The third one was the gene coding for the rRNA methyltransferase RsmD (Fig. 4), which methylates the guanine 66 of the 16S ribosomal RNA during ribosome assembly. The effect of this mutation (rsmD_42) is unclear but is likely to result in a loss-of-function mutation, as the mutation consists of a 1 nt deletion early in the gene.

### Fitness Effects

To estimate the fitness effects of the mutations occurring in the experiment, clones were isolated from populations and whole-genome sequenced. Their growth was compared to their ancestors' growth in both host cell types (Mϕ or *Ac*). Since the 2 fluorescent markers (SYFP2 and dTomato) differ in many aspects (intrinsic effect on organism fitness, brightness, folding time, half-life, etc.), it is not possible to infer fitness differences between 2 clones carrying different fluorescent markers directly from competition experiments. To correct for this, a parallel experiment was run, competing the 2 ancestors, isogenic except for the fluorescent marker. The fitness of the evolved clone competed against the ancestor with the other marker, which can be compared to the fitness of the ancestor with the same marker, competed against the ancestor with the other marker. Two methods to estimate fitness were evaluated: the first assessed growth rates by fitting splines to the growth curves, and the second estimated total growth by comparing the fluorescence in the stationary phase to the fluorescence early in the experiment. The first method proved difficult to reproduce consistently, and the second method, which calculates a competition index (CI), similar to the method used by Ensminger et al. (2012), was preferred.

CI was calculated between the beginning of incubation (0 h) and 64 h of infection. This timeframe represented the beginning and end of infections in our experimental setup (see supplementary fig. S8, Supplementary material online). Since a multiplicity of infection (MOI) of 10 was used, representing 10 bacterial CFUs for 1 host cell, it allowed for only 1 replication cycle. A CI of 1 is equivalent to a neutral effect in terms of fitness between the evolved and the ancestral strain. CI measures the total production of cells during 1 cycle of replication of *Legionella* within its host. It only captures the growth rate indirectly and does not capture differences in how bacteria exit the host cell. The results of competition experiments are shown in Fig. 4.

The *lerC* gene, which encodes a single-domain response regulator involved in modulating the expression of the protein effectors translocated into the host cytoplasm (Feldheim et al. 2018), harbored several independent mutations. Four isolates contained mutations in their *lerC* gene: DA75291i with a nonsense mutation (lerC_46, C46*), DA75299i with a nonsynonymous double mutation (lerC_51, V51G), LG300i with another nonsynonymous mutation (lerC_13, A13D), and DA76737 with a 13 bp deletion (lerC_61). The results of the competition with the ancestors are shown in Fig. 4, while the predicted structure of the mutants is shown in supplementary fig. S7, Supplementary material online. All these isolates also contained streptomycin resistance-conferring mutations: DA75291i (lerC_46, C46*) and LG300i (lerC_13, A13D) also harbor the double mutation RpsL43/RpsD, while DA75299i (lerC_51, V51G) and DA76737 (lerC_61, 13 bp deletion) harbor the other double mutation RpsL88/GroES. The latter 2 isolates, DA75299i and DA76737, also harbor an extra mutation in lpp0833, involved in LPS synthesis. The effects of the mutations are thus compounded in the fitness experiments.

In *Ac* cells, the RpsL88/GroES mutations provided the largest fitness gain (DA75302; CI = 2.34). Adding a mutation in the lpp0833 (involved in LPS synthesis), the fitness was higher than for the wild type but slightly lower than the latter mutant (DA75735; CI = 2.08). The further addition of the 13 bp deletion in LerC (lerC_61) results in a fitness similar to the wild type (DA75737; CI = 0.91), while the addition of the nonsynonymous lerC_51 mutation resulted in a substantial fitness drop (DA75299i; CI = 0.40), suggesting that the net effect of both LerC mutations is a large fitness loss. On the other hand, the RpsL43/RpsD (accompanied by the IolE mutation) yielded a very slight fitness gain (DA76739; CI = 1.11). Removing the IolE mutation but adding the nonsynonymous lerC_13 mutation resulted in a very similar fitness (LG300i; CI = 1.19), while the addition of the nonsense lerC_46 mutation resulted in an increased fitness drop (DA75291i; CI = 0.49) (Fig. 4). Unexpectedly, although mutations in the LPS synthesis cluster were widespread in populations exposed to *Ac*, the largest fitness increase appears to be provided by the RpsL88/GroES mutations.

In Mϕ, the mutations had, in general, a weaker effect, and most tested mutations resulted in a slight fitness decrease. The CI values were, on average, between 0.75 and 1 (with one exception) and showed a greater variance than in *Ac*, making comparisons less reliable. One isolate, DA76737, which has the 4 mutations (RpsL88/GroES, lpp0833, and lerC_61) that are fixed in 2 lineages (Mϕ_A and Mϕ_E), had a markedly lower fitness compared to the ancestral type, with an average CI of 0.52. This value can be compared to the CI of DA76735, which has the same mutations but lacks the nonsynonymous lerC_61 mutation (CI = 0.85). Despite the ubiquity of mutated LerC sequences in populations exposed to Mϕ, no LerC mutants showed increased fitness in Mϕ, at least in the experimental setting used here.

## Discussion

In this study, we experimentally evolved *Legionella pneumophila* in the presence of one of its natural hosts, the amoeba *A. castellanii* (*Ac*), and of the human macrophage-derived cell line U937 (Mϕ) to identify host-specific genetic adaptations. We passaged *L. pneumophila* separately in each host and switching between them, twice a week for a year. In the populations exposed to the amoebal host, we found that the LPS synthesis cluster was probably under selection, whereas in the populations exposed to the human macrophage-derived cell lines, LerC, a protein playing a major role in regulating the temporal expression of protein effectors, harbored potential adaptive mutations. We also found that most mutations occur in mutational hotspots, presumably as a result of the high recombination rates in *L. pneumophila*.

### Host-Specific Adaptations in the LPS Synthesis Cluster and in the LerC Response Regulator

Most *L. pneumophila* populations exposed to *Ac* displayed mutations in genes of the LPS synthesis cluster. The *Legionella* LPS synthesis cluster contains ca. 30 genes (lpg0779-lpg0746 in the Philadelphia strain; lpp0812-lpp843 in Paris) (Thürmer et al. 2009). The genes that were mutated in our evolution experiment (lpp0831, lpp0832, lpp0833, and lpp0835) correspond to ORF 11, 10, 9 and 7, respectively, and belong to the serogroup 1 (SG1)-specific region of the LPS gene cluster (Mérault et al. 2011; Petzold et al. 2013) (Fig. 3). Whereas lpp0831, lpp0832, and lpp0833 have homologs in other serogroups (albeit with low similarity), lpp0835 is found only in SG1 and has been proposed as a marker for that group (Mérault et al. 2011). The function of the heavily mutated lpp0833 (lpg0768) is not fully understood. Still, it is homologous to a sialic acid synthetase function (neuAc, neuB family) and is presumably involved in late modifications of the O-antigen (Petzold et al. 2013). The 1 nt deletion in lpp0833 (lpp0833_182) leads to an out-of-frame mutation after 182 nucleotides (of >1,000), resulting in a very truncated protein. Although functional studies would be required to firmly establish the effect of the truncation, it likely results in a nonfunctional protein.

The fact that 8 distinct mutations were found in the LPS operon, affecting only the populations passaged in *Ac*, strongly suggests that these are adaptive and beneficial for *Legionella* when infecting the *Ac* strain used in this experiment. The isolate with a 1 nt deletion (lpp0833_182) indeed showed a very strong advantage in competitions in *Ac*. However, this isolate also carries the RpsL88/GroES genotype, which itself provides an important advantage. It is thus difficult to separate the effect of the 2 genotypes. Further research into the function of these genes in different hosts might uncover the relationships between LPS and host specificity, but a reasonable hypothesis is that the LPS obtained in the evolved *L. pneumophila* clones is different to the one in the wild-type. The modified LPS thus potentially lowers the immune response of *Ac*, providing an advantage for this particular *Legionella* strain.

The response regulator LerC is another gene carrying multiple distinct mutations (namely 4), 2 of which are fixed in 5 populations at the last time point. LerC is involved in the network of regulation of effector proteins (Feldheim et al. 2018). The primary regulation of the expression happens mainly through 4 distinct 2-component systems (TCS), which regulate over 100 effectors together (Segal 2014). Among these, PmrAB directly regulates over 40 effectors (Al-Khodor et al. 2009), while LetAS regulates another set of effectors via the small RNAs RsmY and RsmZ (Sahr et al. 2009). LerC was found to be a connector between these 2 systems: it is activated by PmrAB and inhibits LetAS (Feldheim et al. 2018). The repression of LetAS is growth phase-dependent, as PmrAB is activated under the exponential growth phase, favoring cell divisions inside the *Legionella*-containing vacuole (LCV). At some point, late in the exponential phase, PmrA stops activating the expression of LerC. LerC thus stops inhibiting the activation of LetA by LetS. Activating LetA, via RsmY/Z, triggers the cells to enter stationary phase (Feldheim et al. 2018).

In the study describing the function of LerC (Feldheim et al. 2018), the authors noticed that knockout mutants of *lerC* resulted in various colony types where one of the types took over after a few passages. Sequencing the new dominant type revealed, in addition to the *lerC* mutation, mutations in either *letA* or *letS* that resulted in nonfunctional versions of the proteins. Although we found several nonfunctional versions of LerC in our experiment, *letA* and *letS* were not mutated in any of our strains. However, *lerC* mutants were grown on solid medium by Feldheim et al., while our lineages were grown in-host only. This indicates that repression of LetAS could be necessary for growth on solid medium. It should also be noted that Feldheim et al. used *L. pneumophila* JR32, derived from Philadelphia-1, which is separated by ca. 70,000 SNPs from the Paris strain used here (David et al. 2016; Wee et al. 2021); the regulation of effector secretion might be different between the 2 strains and could explain why we were able to obtain LerC mutants without the compensating *letAS* mutations.

Mutations in LerC appeared to be generally deleterious in *Ac*. Indirect evidence is provided by the absence of any LerC mutant in the populations exposed to this host only. However, the absence of LerC mutants in these populations could also be the result of clonal interference: other mutations might have had a higher selective value and prevented fixation of LerC mutations in these conditions. Competition experiments also support the deleterious nature of LerC mutants exposed to *Ac*: compared to the strain with the same background except LerC, the LerC mutants had a lower CI. One strain with the wild-type LerC (DA76735) had a CI of 2, while 2 other strains harboring 2 different LerC mutations (DA76737 with the lerC_61 deletion; DA75299i with the nonsynonymous V51G mutation) had much lower CIs, <1 and <0.4, respectively. The comparisons for the other 2 mutants are more complicated to interpret, as there were no isolates with the exact same background. Still, the mutant with the lerC_46 nonsense mutation (DA75291i) had a CI of 0.49, more than half of the counterpart (DA76739), which had the wild-type LerC (CI = 1.11) but also another mutation in IolE. The last LerC mutant (LG300i), carrying lerC_13, a nonsynonymous mutation (A13D), had, in fact, a slightly higher CI (1.19) than DA76739 (1.11). In summary, the distribution, diversity and competition results show that mutations affecting LerC are deleterious for the survival and reproduction of *L. pneumophila* in *Ac*.

On the other hand, the mutations in LerC are likely to be adaptive in human macrophage-derived cells: all 6 populations passaged exclusively in Mϕ acquired a mutation in the *lerC* gene, which got fixed in four of them. Two populations passaged in alternation in Mϕ and *Ac* cells showed mutations in LerC, of which one was fixed. The expected advantage in Mϕ was only partially confirmed by our competition experiments. The comparison with the wild-type strain is complicated because the LerC mutants in our isolate collection all have additional mutations. The most widespread mutation (lerC_61) had a lower CI (0.52) than its counterpart without (0.82), which is unexpected. Conversely, the isolate with the lerC_51 mutation (DA75299i), present in most of the Mϕ_C and Mϕ_D populations, had a higher CI than its counterpart without (DA7535). The fitness for the other 2 LerC isolates (lerC_46 and lerC_13; also harboring the RpsL43/RpsD mutations) is again challenging to evaluate, as there are no isolates with only the RpsL43/RpsD mutations. However, compared to the RpsL43/RpsD isolate with an IolE mutation, the lerC_46 and lerC_13 isolates have a slightly higher CI. In summary, the pervasive presence of LerC mutants in the populations passaged in human macrophage-derived cells strongly suggests that the wild-type LerC protein is deleterious to *L. pneumophila* Paris when infecting these cells.

In summary, although the results of the competition experiments are somewhat inconclusive, the pervasive presence of LerC mutations in populations passaged in Mϕ-like cells suggests that they are likely advantageous for growth in these cells. On the other hand, the absence of mutations in populations passaged only in *Ac* suggests that such an advantage does not extend to these host cell types. In fact, our competition experiments clearly suggest that mutations in LerC are deleterious for growth in *Ac*. The variety of mutations found in the lineages exposed to macrophage-like cells, including two that significantly shorten the protein or completely suppress its expression, suggests that a loss or reduction of the LerC function would be beneficial in the human host. The fact that the LerC mutants are also found in lineages where *Legionella* was alternated between the 2 hosts suggests that the positive selective value in macrophage-like cells is large enough to overcome the negative selective values in *Ac*. Thus, LerC is a good candidate for a gene whose loss confers a host-specific advantage, in this case in human cells.

### An Intergenic Mutation Between an Ion-Transport Operon and a Strain-Specific Gene

One single intergenic mutation was found to be fixed, upstream of 2 genes, lpp2016 and lpp2017 (Fig. 1). The former gene, lpp2016, has no described function and appears to be strain-specific in *L. pneumophila*, while the latter is involved in ion transport. From sequence analysis only, it is not possible to determine whether this mutation would yield a selective advantage. However, it is possible that this mutation interferes with the regulation of one or both of the downstream genes, since potential cis-regulating elements for both these genes would be located in this region. It is also possible that this intergenic space, unusually large (ca. 350 nt), might encode a small, noncoding RNA (ncRNA), which might have regulatory or even virulence functions. To the best of our knowledge, this specific intergenic region has not been shown to contain an ncRNA (Sahr et al. 2012). Recently, *L. pneumophila* has been shown to express and translocate sRNAs into its eukaryotic host, where it regulates the host innate immune response (Sahr et al. 2022).

### Potential Recombination Hotspots Account for a Large Part of the Mutations

*Legionella* genomes are known to experience extensive homologous recombination (David et al. 2017). Early studies estimated that the recombination (r) and mutation (m) rates should be similar (r/m = 0.9) (Coscollá and González-Candelas 2007; Vos and Didelot 2008). However, later estimates based on larger datasets suggest a much higher recombination rate, with the vast majority of SNPs (96% to 99%, depending on the groups) arising from recombination (David et al. 2017). In our experiment, recombinations could occur intragenomically or intergenomically from DNA acquired by transformation from other *Legionella* within the same vacuole. In the first case and if the homologous recombination occurs between similar but nonidentical regions, it would result in genome rearrangements and in mutation clusters at the recombination sites. In the latter case and if the recombination occurs between homologous segments, the gained alleles would only affect allelic frequencies in the population, but not create mutation clusters. Here, we identified 5 groups of homologous regions that harbor mutation clusters. These regions might represent hotspots of intragenomic recombinations: 11 homing endonucleases, an ankyrin repeat-containing protein (lpp1100), 1 tetratricopeptide repeat (TPR) protein, a repeated protein containing a domain of unknown function (DUF1566), and 3 occurrences of a larger segment containing a transposase and a restriction endonuclease type II-like (DUF559). The 2 groups of hotspots present in different locations of the genomes (homing endonucleases and DUF559) showed further evidence of recombinations. Indeed, out of the 61 long-range structural variants detected in our analysis, almost half (44%) involved recombinations between pairs of these hotspots (supplementary fig. S6, Supplementary material online).

The hypothesis that mutation clusters found in our experiment were created by intragenomic recombinations is supported by the results of the variant caller, breseq. The structural variants are identified based on conservative criteria, established and tested on the datasets coming from the Lenski *Escherichia coli* long-term evolution experiment (LTEE) (Deatherage et al. 2015). However, none of the variants detected here were experimentally confirmed, and we cannot exclude that they are, at least partially, the result of alignment errors or other technical artifacts. Given that such a high level of recombination has never been demonstrated in *L. pneumophila*, further experiments are necessary to confirm the results presented here.

The first group of potential recombination hotspots consists of homing endonucleases—found in all domains of life and in viruses—which are known to cause gene conversions between mobile genetic elements (Dunin-Horkawicz et al. 2006). This group of genes has been found to be enriched for in clinical sporadic strains (when compared to environmental strains) in *L. pneumophila* sequence type (ST) 1, suggesting that they contribute to the genetic diversity of ST1, the most prevalent ST (Mercante et al. 2018). The second group is an ankyrin repeat-containing protein (ANK). ANK motifs are typically eukaryotic motifs and are found on many *Legionella* effectors (Habyarimana et al. 2008). This specific protein is not widespread in *Legionellales* and is absent from most *L. pneumophila* strains and from *L. longbeachae* (Gomez-Valero et al. 2011). It has been shown to be highly upregulated in transmission phase (Brüggemann et al. 2006; Jules and Buchrieser 2007). A distant homolog is, however, found in *L. drancourtii* LLAP12 (supplementary fig. S9, Supplementary material online). TPR motifs, the third group of potential recombination hotspots, are involved in protein–protein interactions in eukaryotes and bacteria and in the virulence in *L. pneumophila* (Bandyopadhyay et al. 2012). The fourth hotspot is a nonrepeated protein containing a domain of unknown function (DUF1566) widely present in *Gammaproteobacteria*. Finally, the fifth group of hotspots comprises 3 occurrences of a larger segment containing a transposase and a restriction endonuclease type II-like (DUF559). The first and fifth groups were recognized as repeated regions (David et al. 2017) and excluded from their recombination analysis. As for the other single-protein hotspots, they were not deemed repeated but were not detected as recombination hotspots.

About half (49.4%) of the mutations identified in our study occur in these 5 groups of mutational hotspots, but none of them was fixed. Most occurred only at low frequency, and only mutations in 3 hotspot groups (lpp1100, GIY-YIG homing endonucleases, transposase/DUF559) reached 25% in frequency. However, the large number of intragenomic recombination events that can be detected probably provides variation in the genome architecture and potentially contributes to the adaptability of *Legionella* genome.

The mutational patterns identified in this study follow the general principles of molecular evolution, for example, displaying about twice as many transitions as transversions. Single-nucleotide polymorphisms generally lower the general G + C content of bacterial genomes (e.g. Hershberg and Petrov 2010; Ely 2021), while higher G + C content is believed to occur mostly through GC-biased gene conversion (gBGC) (Lassalle et al. 2015). However, in our study, the AT to GC mutations unexpectedly accounted for almost half (49.4%) of the mutations altering the GC content, even after removing mutations occurring in recombination hotspots.

The pLPP plasmid in *L. pneumophila* strain Paris (Cazalet et al. 2004) is sparsely found in ST1 strains (Durieux et al. 2019). Strikingly, although the plasmid is not essential, it has been retained in all our lineages. This may be due to a toxin/antitoxin (TA) system on the pLPP plasmid: at least 1 PHD-RelE type TA system on the pLPP plasmid, according to the TADB (Shao et al. 2011) and TAsmania (Akarsu et al. 2019).

### Fitness of Evolved Strains

A puzzling observation in the fitness measurements performed is the discordance between our finding in the serial passage experiments of parallel and fixed mutations, which indicates a strong advantage, versus the lack of an advantage of the evolved clones (observed CI < 1) in competition experiments with the ancestral strains. At present, we do not understand the underlying reason(s) for this discordance; it should be noted that measuring fitness is a complex matter and that in an experiment like the one described here, selection acts at different levels. Compared to experiments in pure culture like the LTEE (Lenski 2017), where the maximum growth rate in the exponential phase is paramount, other aspects of the life cycle of *Legionella* play a role. The time necessary to find a host, the ability to trigger endocytosis, the ability to resist digestion and the efficiency to recruit nutrients to the *Legionella*-containing vacuoles, the time required to exit lag phase and enter exponential phase, and the efficiency to exit the host cells are all important in how many of each genotype make it to the following passage. Thus, the CI method used here is probably too crude to capture all these aspects, possibly explaining the sometimes-discordant results presented above. It is also possible that the fitness changes are nontransitive and that competition experiments of the evolved strains versus the ancestral strain do not adequately capture such dynamics. Additional fitness measurements might help better understand the phenotypic effects of the mutations.

### Comparison With Other Studies

Other studies have identified host-specific mutations occurring within the host. In the first and—to our knowledge—only published evolution experiment with *L. pneumophila*, Ensminger et al. (2012) passaged *L. pneumophila* in mouse Mϕ for a year. This study identified, among others, mutations in the lysine synthesis pathway and in the flagellar regulation gene *fleN*. Surprisingly, there was no overlap between the mutations found in this study and ours. One similarity is in the mutations identified upstream of the *pacS* gene in the former study, respectively upstream of lpp2017 in our study: both genes are involved in transporting inorganic ions (copper in the case of *pacS*, possibly other cations for lpp2017). This lack of overlap might however also reflect the differences between the hosts and *Legionella* strains used in the 2 studies: while we used human cell lines and *Ac* as hosts and *L. pneumophila* Paris, the other study cycled *L. pneumophila* LP01, derived from the Philadelphia-1 strain, cycled in mouse-derived Mϕ. Coincidentally, many lab strains derived from *L. pneumophila* str. Philadelpha-1 (strains JR32, Lp01, and Lp02, among others) were selected on streptomycin, and are thus resistant to that antibiotic (Rao et al. 2013). A comparison with another study identifying mutation occurring in-patient (Leenheer et al. 2023) yielded 1 single match: in this study, the gene *lidB* (lpp2223/lpg2269) was found to be mutated twice independently in different outbreaks, while this gene is mutated in 13% of the population ​Mϕ_A at t2. LidB is an ATP-dependent helicase/deoxyribonuclease protein, possibly involved in replication. However, the evidence in both studies is too sheer to conclude a possible selective advantage of a mutated version of this protein without further functional studies.

### Early Fixed Mutations

An unexpected finding was the presence of multiple, apparently fixed mutations at the first time point (i.e. after 10 to 15 passages), some of which subsequently disappeared (Fig. 1). We first discuss the probabilities of the same mutations appearing independently in various lineages and then the fact that some disappeared despite being apparently fixed.

The analysis of mutation rates and population sizes shows that parallel evolution in our setup is not unlikely. The mutation rates to streptomycin (mutations per cell per generation) measured by fluctuation assays on our ancestral strains (*L. pneumophila* Paris and the 2 fluorescently labeled strains) were ca. 6.5 × 10^−10^. In comparison, mutation rates for bacteria range from 6 × 10^−11^ to 3 × 10^−8^ (Lynch et al. 2023), putting our isolates in the middle. We calculated the probability of the appearance of a mutation providing resistance to streptomycin, given the number of generations and the mutation rates above, assuming no selection. Since streptomycin resistance is often provided by 2 single RpsL mutations (K88R and K43T), the probability of gaining any given mutation is close to the probability of gaining a streptomycin resistance mutation. For an amoeba-exposed or a human cell-exposed population, the probability was 4% and 39% at the first time point (t1), while it reached 23% and 76% at the last time point (t3), respectively. This shows that it is not unlikely that single, identical mutations would appear parallelly in different populations. Given a strong enough selection, these mutations could have been quickly fixed in the population.

However, although other recurring mutations (e.g. in LerC or in lpp0833) are very likely to have occurred independently in different lines, the 2 paired mutations (Rpsl43/RpsD and Rpsl88/GroES), which always occurred together and in the same genetic background (SYFP2 and dTomato, respectively), have a higher likelihood to be the result of a spread of these 2 genotypes very early in the experiment. We had established very rigorous procedures to minimize the risks of cross-contaminations: bacteria were grown in separate flasks and manipulated individually, always in a laminar flow cabinet, using filter tips when pipetting, and applying general sterility precautions when manipulating the strains. These 2 genotypes have rapidly taken over most populations, emphasizing their probable high selective value. Although a possible early contamination across lineages might have caused the spread of the RpsL mutants, we believe that the latter would be the only ones to be spread that way and thus that the conclusions of the paper would not be affected.

To explain that a few apparently fixed mutations disappeared at a later time point, we consider 3 possible explanations: contamination across lineages, reversions, or not deep-enough sequencing. As mentioned above, we have established very rigorous procedures to minimize the risks of cross-contamination and thus favor the latter 2 hypotheses, either that the mutations reverted or that the sequencing missed the presence of the original allele. The apparently high selective value of some of the later mutations might explain these reversions or re-emergence of low-frequency wild-type alleles. An analysis of the reads mapping to the genes encoding the fluorescent markers (SYFP2 and dTomato) shows that in the populations harboring mutations considered fixed, and which therefore should contain only the reads mapping to only one of the fluorescent genes, a few reads of the other gene can be found (supplementary fig. S10, Supplementary material online, see e.g. *Ac*_D or Alt_E). We cannot exclude that some of these reads might come from demultiplication errors in cases where there are 1 or 2 reads. However, at least in the 2 above-mentioned populations, their prevalence is likely too high to be explained by artifacts. This suggests that the population still contains at least low frequencies of both populations, which might explain how apparently fixed mutations might disappear, should a more favorable mutation appear in the genome of the low-frequency genotype. It should be noted, however, that this latter hypothesis is not particularly probable, as these favorable mutations are more likely to occur on the high-frequency genomes.

### High Number of Nonfixed Mutations

A surprising result of this study is the amount of nonfixed mutations: among those present in at least 5% of the population, only 6% (91/1500, Table 2) became fixed. Several factors can explain this observation: high mutation rate, low selection, small population sizes, and extensive clonal interference, which prevents the fixation of mutations with a lower selection value (see e.g. Bailey et al. 2017). The average mutation rate is not remarkably high, as shown by our fluctuation tests (6.5 × 10^−10^; see supplementary Results and Discussion, Supplementary Material online). The strength of selection is difficult to measure directly, but the fact that only 2 out of 91 fixed mutations are synonymous tends to indicate that genetic drift (essential to get neutral mutations to fixations) does not play a large role here. The relatively small bottleneck population sizes (5 × 10⁵ for *Ac* populations, 5 × 10^6^ for Mϕ) would nonetheless let genetic drift play a role. Our results are comparable with a previous evolution experiment conducted with *L. pneumophila* in mouse Mϕ (Ensminger et al. 2012): the authors set up similar bottleneck population sizes as we did for *Ac* populations. They obtained on average 3.5 fixed mutations per populations, while we reached a similar number for populations passaged with Mϕ (3.6), but a lower one for populations passaged with *Ac* (1.7). The authors identified several mutations which transiently increased in frequency, as a result of clonal interference. We show that there is a substantial amount of recombination going on in our experiment, which would mitigate clonal interference by allowing beneficial mutations to gather in a single genome. However, here we only demonstrate that recombinations occur intra-genomically, not that organisms actually exchange alleles, which would require some functional horizontal gene transfer mechanism. In summary, clonal interference might also limit the fixation of beneficial alleles in this experiment, despite recombinations occurring in *L. pneumophila* genomes.

## Conclusion

In summary, the exact role, fate, and history of the mutations that occurred in the 18 lineages of *L. pneumophila* investigated here remain to be established. However, for both hosts, we identified potential adaptive, host-specific mutations. Lineages exposed to the amoebal host *A. castellanii* harbored mutations in the host-specific part of the LPS synthesis cluster. Lineages exposed to the human macrophage-derived cells displayed mutations in the LerC protein, a protein regulating the expression of protein effectors, potentially highlighting the different temporal regulation of protein effector secretion in the human host compared to *L. pneumophila*'s natural hosts. These results also show that experimental evolution can reveal bacterial genes specifically involved in the adaptation to specific hosts.

## Materials and Methods

### Bacterial Strains, Host Cell Culture, and Media

*Legionella pneumophila* Paris was cultured in charcoal yeast extract (CYE) [1% ACES, 1% yeast extract, 0.2% charcoal, 1.5% agar, 0.025% Iron {III} pyrophosphate, 0.04% L-cysteine, pH 6.9] plates or ACES yeast extract (AYE) [1% ACES, 1% yeast extract, 0.025% Iron {III} pyrophosphate, 0.04% L-cysteine, pH 6.9) broth at 37 °C, unless otherwise stated. *Legionella* GVPC selective supplement (Oxoid), or 0.5 mM IPTG, was added to the media when necessary. The *L. pneumophila* strains were tagged with an SYFP2 or dTomato fluorescence gene under an IPTG-inducible promoter. A *L. pneumophila* Paris mutant harboring a deletion in the *dotA* gene was used as a negative control in infection experiments, as this strain is not able to replicate in host cells (Descours et al. 2017).

*A. castellanii* (*Ac*) strain Neff (ATCC 30010) was cultured in Peptone Yeast Glucose (PYG) medium (ATCC 712 medium) in culture flasks at 30 °C. For infections, *Ac* was harvested, pelleted, and resuspended in LoFlo medium (Formedium, Norfolk, United Kingdom), which does not support *Legionella* growth.

Human monocyte-like U937 cells (ATCC-CRL-3253, abbreviated Mϕ) were maintained in RPMI1640 + GlutaMAX (Gibco) supplemented with 10% heat-inactivated fetal bovine serum (FBS) (Gibco) and 1% Penicillin-Streptomycin (Pen-Strep; Gibco), in a 37 °C incubator with 5% CO_2_. To induce differentiation into macrophage-like cells, the Mϕ culture was centrifuged at 200 × *g* for 5 min, resuspended in growth medium containing 50 ng ml^−1^ of phorbol 12-myristate 13-acetate (PMA), and incubated for 48 h. The medium was replaced, and cells were incubated for 48 h. Before infection, cells were resuspended in RPMI 1640 without phenol red (Gibco) supplemented with 10% heat-inactivated FBS and 1% GlutaMAX (Gibco). This medium also does not support *Legionella* growth.

### Passaging

*Legionella pneumophila* SYFP2 and dTomato, patched on CYE for 48 h, were resuspended in dH_2_O, the OD_600_ was measured, and the cultures were diluted before being mixed in equal proportions. Host cells were prepared as above; 2 × 10^6^ cells of *Ac* or Mϕ were seeded in 6 T25 flasks and challenged at an MOI of 0.25 (*Ac*) and 2.5 (Mϕ). After inoculation, flasks were incubated at 30 °C or 37 °C for 3 d. Flasks were vortexed at the end of the infection period, and an aliquot of the culture was collected. These aliquots were centrifuged for 5 min at 200 × *g* to pellet the host cells. The endpoint concentration of *L. pneumophila* for each infection was estimated by measuring the OD_600_ and calculating the CFU based on a standard curve (OD_600_ vs. CFU/ml) made for this purpose. The volume of bacteria needed to infect the hosts at the same MOIs was used to inoculate fresh host cultures. Thus, each new passage of *Ac* was inoculated with 5 × 10⁵ CFUs, whereas the macrophage lineages were inoculated with 5 × 10⁶ CFUs. To confirm there was no contamination of the cultures, 10 µl of the cultures was plated on LB agar and CYE plates. *Legionella pneumophila* was passaged in this manner every 3 to 4 d. At every fifth passage, glycerol stocks were made by collecting all the infection supernatant (after pelleting the hosts) and centrifuging at 7,000 × *g* for 15 min to pellet the bacteria. The resulting pellet was resuspended in AYE with 50% glycerol and kept at −80 °C.

### Measure of Growth Rates

To isolate individual clones, frozen stocks of the selected populations were streaked on CYE + GVPC plates and incubated for 72 h. The bacterial lawn was scraped and resuspended in ultrapure water, and serial dilutions were made and plated on CYE + IPTG plates to obtain single colonies. Two colonies of each population were restreaked, and glycerol stocks were made.

The ancestor and evolved strains' fitness assays were performed in AYE broth and hosts. For growth in broth, a 24 h patch of each colony was resuspended in AYE, and the density (OD_600_) was measured and adjusted to 0.1 (ca. 2 × 10^8^ CFU/ml) with AYE + IPTG. Each culture was aliquoted in triplicate into a black 96-well plate; the growth rate was tracked by measuring the OD_600_ and fluorescence of SYFP2 (excitation, 508 nm; emission, 555 nm) or dTomato (excitation, 554 nm; emission, 635 nm) every 30 min for 24 h using a Tecan Spark 10M equipped with a monochromator. For in-host growth assays, bacteria and hosts were prepared as before, but here, the 1 × 10^5^ host cells were seeded per well in a black 96-well plate and challenged at an MOI of 50. The infection media was supplemented with 0.5 mM IPTG. Intracellular replication was tracked by measuring the fluorescence every 30 min for 72 h, as described above.

### Competition Assays

Competition assays were performed in Mϕ and *Ac* using a similar protocol to the one used by Ensminger et *al.* (2012). For in-amoeba competitions, evolved strains to be assayed were streaked onto CYE plates with IPTG (1 mM) and incubated at 37 °C for 3 d. Biomass was taken from the lawn created by each strain and resuspended into either LoFlo medium (Formedium, Norfolk, United Kingdom) with 1 mM IPTG (for infections in amoebae) or in RPMI without phenol red and with 1 mM of IPTG (for infections in Mϕ). These solutions were diluted to an OD_600_ of 0.50 (around 5 × 10⁸ CFU/ml) with a Tecan Spark 10M plate reader. Each tested strain was mixed in equal parts with the ancestral strain (*L. pneumophila* Paris) with the other fluorescent tag: dTomato-tagged ancestral strain with SYFP2-tagged evolved strains and SYFP2-tagged ancestral strains with dTomato-tagged evolved strain. The solutions were serially diluted down to 1/10, and a volume, depending on host species, was transferred to the corresponding wells of 24-well plates containing either *Ac* or Mϕ cells.

For competitions in amoebae, 200 µl of the mix of bacteria was incubated with 5 × 10⁵ *Ac* cells in 2 ml of LoFlo medium with 1 mM of IPTG to reach a multiplicity of infection (MOI) of 10. This MOI was chosen as it exceeds the minimal level of fluorescence detectable by the plate reader at the beginning of the assay. The plates were incubated at 30 °C.

For competitions in Mϕ, the host cells were seeded in the wells at 2.5 × 10⁵ cells per well, adding 100 µl of mixed evolved/ancestral bacterial strain solution to reach a MOI of 10. RPMI without phenol red but with 1 mM of IPTG was used as medium for the infection of the bacterial inoculum solutions. Finally, the 24-well plates were incubated at 37 °C with 5% CO_2_.

For both competitions, the ancestral dTomato- and SYFP2-tagged *L. pneumophila* were also competed on each plate as a reference. All competitions were done in 5 replicates for each pair of strains tested. The following controls were also performed on each plate: growing *Ac* or Mϕ cells only (blank control), ancestral SYFP2-tagged strain and *Ac* (SYFP2 positive control), ancestral dTomato-tagged strain and *Ac* (dTomato positive control), and Δ*dotA* mutant (infection-negative control, infection-defective). The *ΔdotA* was used as infection-negative control, and CFU/ml counts were measured at the competition experiment's beginning and end.

The fluorescence was measured with the Tecan Spark 10M spectrofluorometer at an excitation/emission wavelength of 508/555 and 554/635 nm to measure SYFP2 and dTomato fluorescence, respectively. Fluorescence was measured at 0, 40, and 64 h postinfection. In our experimental setup, these time points correspond to the beginning of the infection, the start of the exponential phase, and the beginning of the postexponential growth phase. The time points were determined by measuring growth curves and optimizing the conditions (see supplementary fig. S8, Supplementary material online). Fluorescence data was used to calculate a competition index (Ensminger et al. 2012). The fluorescence measurements were blanked with the fluorescence values of the *Ac*-LoFlo or Mϕ-RPMI control wells. Competition index (CI) is calculated as the ratio of the fluorescence of the mutant (evolved) strain to the fluorescence of the wild type (ancestor) strain at t1, divided by the same ratio at t0:


$$\mathrm{CI}\;=\;[F{\left(\mathrm{mut}\right)}_{t1}/F{\left(\mathrm{WT}\right)}_{t1}]/[F(\mathrm{mut}{)}_{t0}/F(\mathrm{WT}{)}_{t0}]$$

where

*F*(mut)_t1_ = Fluorescence of mutant at end time point (t1);

*F*(WT)_t1_ = Fluorescence of wild type at end time point (t1);

*F*(mut)_t0_ = Fluorescence of mutant at beginning time point (t0);

*F*(WT)_t0_ = Fluorescence of wild type at beginning time point (t0).

We normalized the CI data of each strain tested by dividing their CIs by the average CI of the SYFP2-tagged ancestor against the dTomato-tagged ancestor. This allowed us to compare CIs across plates. The competition index reflects the ratio of the number of mutant cells to the number of ancestral cells produced between t0 and t1.

### Fluctuation Assays to Determine Mutation Rates

Fluctuation assays were carried out according to the recommendations of Lang (2018) and Rosche and Foster (2000). Briefly, the tested strains (*L. pneumophila* Paris WT and the 2 SYFP2 and dTomato-tagged mutants) were inoculated in AYE broth and diluted to reach an OD_600_ of 0.50, corresponding to ca. 5 × 10^8^ CFU/ml. The solutions were diluted 1/1,000 by adding 5 µl of this solution to 5 ml of fresh AYE and vortexed. From these, 100 µl were used to inoculate 2 ml of AYE into 50 ml Falcon tubes. This was repeated 72 times for each strain tested. The tubes were incubated at 37 °C for 3 d. After growth, 150 µl of the solution was used to count CFU/ml numbers using the serial-dilution spot plate method (Wang et al. 2017). The remaining solution was centrifuged at 6,000 × *g*, and most of the supernatant was removed. The bacterial pellet was resuspended in the remaining supernatant and plated on CYE containing 6 µg/ml streptomycin, around 8 × the minimum inhibitory concentration (MIC). The plates were incubated for 3 to 4 d, and total and resistant colonies were counted. Mutation rates were calculated using the web-based Fluctuation AnaLysis CalculatOR (FALCOR) (Hall et al. 2009). The MSS maximum likelihood method was used to calculate mutation rates using the “Group all data” option.

### Identification of *rpsL* Mutations in Streptomycin-Resistant Strains

To survey the various mutations contributing to streptomycin resistance, we picked 40 streptomycin-resistant colonies from the fluctuation assay and sequenced their *rpsL* gene, which confers resistance mutations to streptomycin (Zhu et al. 2015). This was done by amplifying a 300 bp segment of the *rpsL* gene using the following primers: forward 5′-AAGAAAGCCTCGTGTGGACG-3 and reverse 5′-TCGGTCGTTCACTCCTGAAG-3′. The DreamTaq Green PCR Master mix (Thermo Fisher Scientific) was used to amplify the gene, and the manufacturer's instructions were used for PCR reactions with an annealing temperature of 59.6 °C and 25 cycles. PCR reactions were checked on 1% agarose gel and then purified using the GeneJET DNA Cleanup Micro kit (Thermo Fisher Scientific). The purified fragments were sent for Sanger sequencing to identify mutations.

### Streptomycin Susceptibility Testing

The level of streptomycin resistance of the ancestral strains and evolved isolates was evaluated using an E-test on CYE + IPTG agar plates. The minimum inhibitory concentration (MIC) was recorded as the lowest antibiotic concentration at which the zone of inhibition intersected the E-test strip; for many isolates, there was a zone with a haze of growth around the strip. Here, the MIC was read the same way, but the MIC at the start of the hazy zone was also recorded (supplementary fig. S11, Supplementary material online).

The selected *Legionella* strains were grown on CYE + GVPC plates for 48 to 72 h; a loop of bacteria was suspended in dH_2_O, and the density was adjusted to OD_600_ = 0.05 (ca. 1 × 10^8^ CFU). Then 1 ml of the *Legionella* suspension was added to 5 ml of 0.5% (soft) agar solution; the mixture was gently vortexed to avoid the formation of bubbles and poured onto a CYE + IPTG agar plate. The plates were left to solidify for 10 to 15 min before applying the streptomycin E-test strip (AB-BIODISK, bioMérieux). The plates were then incubated at 37 °C, and the results were taken at 48 h.

### Population and Clone Sequencing

To identify the mutations that arose from the evolution experiment in the different lineages, we sequenced populations at passages 10 and 65 for the *Ac* lineages, passages 10 and 49 for the alternating lineage, and passages 15 and 38 for the Mϕ lineages (supplementary table S1, Supplementary material online). All replicates from each lineage were sequenced. All clones involved in competition experiments were also sequenced to confirm that other mutations did not occur (supplementary table S3, Supplementary material online).

Briefly, the frozen stocks of the different lineages were plated on CYE agar plates and incubated at 37 °C for 3 d. After incubation, the bacterial growth was scraped from the plate and suspended in 5 ml of distilled water. The cultures were centrifuged at 6,000 × *g* for 5 min, and the supernatant was removed. Centrifugation and supernatant removal were repeated. The DNA was extracted using the DNeasy blood and tissue kit (Qiagen), using the pretreatment for Gram-negative bacteria, following the manufacturer's instructions, with the following exceptions: the lysis incubation period was increased to 3 h, 4 µl of 10 mg/ml of RNase A was used, and the DNA was eluted in water. Next, the DNA samples were prepared for barcoding and sequencing using the Nextera XT kit from Illumina. The manufacturer's instructions were followed to prepare the libraries.

### Variant Calling

Reads were processed for quality using fastp version 0.23.2 (Chen et al. 2018). Adapters were trimmed from the reads using the –detect_adapter_for_pe option, and the –overrepresentation_analysis, –correction, and –cut_right options were used for quality trimming. Trimmed reads were deposited at ENA under study accession number PRJEB82630. Variant calling using the clean and processed reads was done using breseq version 0.36.1 (Deatherage and Barrick 2014). In brief, breseq calculates a Bayesian score in which all disagreements with the consensus at each position derive only from sequencing errors. It also filters out potential mutations with a strand bias, a quality bias, and positions in homopolymeric stretches. Finally, it only calls variants with at least 5% frequency in the population. The –polymorphisms-prediction option was used for population sequencing for variant calling. To control for false positives, we mapped the reads obtained from single colonies or the fluorescently tagged ancestors, using the polymorphism prediction. We find that Breseq identifies a few mutations, namely, 15 in the SYFP2 clone and 7 in the dTomato one, with low frequencies (on average 9.8% and 7.5%, respectively) (supplementary figs. S2 and S3, Supplementary material online). These detected mutations may be false positives or mutations that arose during the growth of the colony used for sequencing.

Breseq also identifies potential new junctions (corresponding to recombinations when analyzing single genomes) by analyzing reads which map to 2 different locations of the reference genome. Briefly, breseq detects reads that map to one part of the reference on one end of the read and to another part of the reference on the other end (reads referred to as “split reads”). Potential junctions are retained only (i) if some split reads map to both strands of the junction, (ii) if some split reads mapping to the junction extend at least 14 bp into each side of the reference, (iii) if some reads mapping to each strand of the junction extend at least 9 bp into each side of the reference, and (iv) if reads mapping to the junction extend at least 3 bp on each side (Deatherage et al. 2015). To focus on recombinations occurring outside of tandem repeats, junctions where ends map closer than 5 kb from each other were discarded.

One population where the hosts were alternated (Alt_D) displayed a large excess of mutations at passage 10 (t1): while the median number of mutations is 28.5 per population, Alt_D displayed 989 mutations. A transient contamination from another *L. pneumophila* strain could be identified in this population. The mutation frequency distribution showed a clear peak around 6% (supplementary fig. S12, Supplementary material online). In this population, all mutations with a frequency lower than 20% were filtered out, thus retaining only 15 mutations, none of which were fixed.

Mutations occurring in regions containing many tandem repeats were filtered out.

For coverage analysis of the reads mapping to the genes encoding fluorescent genes, the trimmed reads were mapped to the sequence of either gene using bowtie2 v2.4.4 (Langmead and Salzberg 2012), removing bad reads with –qc-filter.

### Statistical Analysis and Visualization

All statistics and most figures in this contribution were performed in R (R Development Core Team 2024), using the tidyverse (Wickham et al. 2019), particularly the ggplot2 package (Wickham 2009). Gene maps were produced with the R package genoPlotR (Guy et al. 2010).

### Structure Prediction of the LerC Mutants

Alternative gene prediction of the *lerC* mutants (lerC_46 and lerC_61) was performed by inputting the mutant sequence of the gene in prodigal 2.6.3 (Hyatt et al. 2010), with default options. Structures were predicted using AlphaFold3 (Abramson et al. 2024) and then superimposed using PyMol v2.5.4 (Schrödinger, LLC 2015).

## Supplementary Material

msaf161_Supplementary_Data

## Data Availability

Population sequencing data is available at ENA under study accession number PRJEB82630.
