# Supplementary material for: Host-Specific Adaptation of Legionella pneumophila to Single and Multiple Hosts
Source: Mol Biol Evol. 2025 Jul 3;42(8):msaf161. doi: 10.1093/molbev/msaf161 (PMC12308824; doi:10.1093/molbev/msaf161)
Supplement: msaf161_Supplementary_Data [file msaf161_supplementary_data.pdf]

Host-specific adaptation of *Legionella pneumophila* to single and multiple hosts – Supplementary Material

Anaísa B. Moreno[1,†], Kiran Paranjape[1,†], Martina Cederblom[1], Elisabeth Kay[2], Christian Dobre-Lereanu[1], Dan I. Andersson[1], and Lionel Guy[1,*]

[1] Department of Medical Biochemistry and Microbiology, Science for Life Laboratories, Uppsala University, Uppsala, Sweden
[2] Centre International de Recherche en Infectiologie, Lyon, France

[†] These authors contributed equally to the study.
* Corresponding author (lionel.guy@imbim.uu.se)

Supplementary Results and Discussion

Two pairs of mutations in ribosomal protein and chaperonin genes are mutually exclusive

The distribution of mutations showed two distinct patterns of mutations, both of which were present under all three conditions (Figure 2). In one (referred to as RpsL43/RpsD), the 30S ribosomal protein S12 RpsL was mutated at position 43, where the lysine is replaced by a threonine (K43T), while the 30S ribosomal protein S4 RpsD is mutated at position 205, where a serine replaces a tyrosine (S205Y). In the other pattern (RpsL88/GroES), RpsL has another mutation, where the lysine at position 88 is replaced by an arginine (K88R), while the co-chaperone GroES has a mutation at position 45 (A45T). Every population harbored one or the other pair of mutations, or a mix of both, at the last time point. Strikingly, these two pairs of mutations were never seen together in a single isolate. In the four cases where both patterns were present (*Ac*_D, *Ac*_F, Alt_A, and Alt_C; Figure 2), the frequencies of the two mutations within the pattern were very similar, and the sum of the two patterns roughly equals one. An inspection of the reads spanning both positions in the *rpsL* gene revealed that no single read displayed both mutations but that all reads carried one.

The two mutations in RpsL (K43T and K88R) have previously been shown to provide resistance to streptomycin in *L. pneumophila* (Rao et al. 2013). In both cases, we determined the minimal inhibitory concentration to be over 1024 µg/ml. For both mutations, a halo zone was observed on plates (Supplementary Figure 11, but the halo was larger in the K88R-carrying mutant (down to ~96 µg/ml) than in the K43T-carrying mutation (down to ~768 µg/ml).

As a result, the gene *rpsL*, encoding the 30S ribosomal protein S12, was mutated in all populations, conferring streptomycin resistance to *L. pneumophila*. Two distinct mutations (at positions 43 and 88) were identified, and each was accompanied by another mutation. The RpsL43/RpsD double mutation is found in 11 out of 18 lineages and is fixed in 7. The other double mutation conferring streptomycin resistance, RpsL88/GroES, is equally widespread and is also found in 11 out of 18 lineages and fixed in 7. One isolate with the second genotype (Rpsl88/GroES; DA75302) showed a strong advantage in competitions in amoebae but a slight disadvantage in macrophages (Mϕ) (CIs of 2.34 and 0.76, respectively) (Figure 4). None of our isolates had only the RpsL43/RpsD genotype, but one (DA76739) had these two mutations and an extra G241V non-synonymous mutation in the IolE protein, involved in myo-inositol metabolism. This isolate showed a similar trend, but not as pronounced, as the RpsL88/GroES mutant – it grew slightly better than the wild type in amoebae but worse in Mϕ (CI values of 1.11 and 0.73, respectively) (Figure 4).

Mutation rates and diversity of streptomycin resistance-conferring mutations

To investigate the likelihood that the same mutations (in particular, the ones conferring resistance to streptomycin) occur many times in parallel, we measured mutation rates to streptomycin using fluctuation assays for the three ancestral strains (*L. pneumophila* Paris, and both SYFP2- and dTomato-tagged descendants). The results indicated that the mutation rates were low for all three strains (see Supplementary Table 2), ranging from 5.5×10^{-10} to 7.6×10^{-10}. Considering an average mutation rate of 6.5×10^{-10} and that the bacterial population size is 5×10^{5} and 5×10^{6} for the experiments with *A. castellanii* (*Ac*) and Mϕ cells, respectively, there is a 3.3×10^{-4} and 3.3×10^{-3} probability that a mutation conferring resistance to streptomycin will appear in one cell at each generation. At t1, which corresponds to 128 and 113 generations (Supplementary Table 1), the probability that streptomycin resistance would have occurred at least once in any given *Ac* and Mϕ population reaches 4% and 30%, respectively. At t3 (798 and 444 generations), the probabilities were 23% and 76%, respectively. However, the streptomycin resistance was present in half the *Ac* populations, 5 out of 6 Mϕ populations at t1, and in all populations at t3, exceeding the calculated likelihoods, indicating that streptomycin resistance might have introduced by contamination.

To investigate whether the only two RpsL mutations we obtained in our evolution experiment (K43T and K88R) were representative of all streptomycin-resistance conferring mutations, we sequenced the *rpsL* gene of 40 streptomycin-resistant isolates obtained from the fluctuation assays. Although six other mutations were identified, K43T (11 mutations) and K88R (21 mutations) accounted together for 80% of the mutations found in these isolates (n = 40) (Supplementary Figure 13).

Adaptation to experimental growth conditions

In addition to the potentially host-specific adaptations, we identified groups of mutations involved in adaptation to the specific growth conditions of our experiment. Among them are two mutations in the ribosomal protein S12 RpsL (K43T and K88R), known to provide resistance to streptomycin (Funatsu and Wittmann 1972). Spontaneous resistance to streptomycin resistance in *Legionella* is a common phenomenon, e.g. in *L. dumoffii* (Hubber et al. 2017) or in many laboratory descendants of the *L. pneumophila* Philadelphia (Berger and Isberg 1993; Rao et al. 2013). Single-step resistance to streptomycin through mutation of *rpsL* has been documented, among others, in *Escherichia coli* (Funatsu and Wittmann 1972) and *Mycobacterium tuberculosis* (Nair et al. 1993). In both organisms, a mutation in codon 43 (42 in *E. coli*) provides resistance to streptomycin, while mutations in codon 88 (87) are found mostly in *E. coli* (Timms et al. 1992). Both mutations have been identified in spontaneous streptomycin mutants in *L. pneumophila* (Rao et al. 2013). Our *Legionella* lineages were never directly exposed to streptomycin, but early in the experiment, both host cell lines were grown in media containing penicillin-streptomycin to avoid contaminations. The host cells were thoroughly washed

before infection with the *Legionella* lineages, precisely to prevent exposing *Legionella* to the antibiotic. A possible explanation is that, despite washing, streptomycin could accumulate in the host cytoplasm, where it gets in contact with bacteria and increases the selective pressure for streptomycin resistance. Although it is unlikely that the concentrations inside the washed host cells reached the minimum inhibitory concentration (MIC), sub-MIC levels of antibiotics have been shown to select for resistant mutants (Gullberg et al. 2011).

Each of the two RpsL mutations described above is accompanied by another mutation in almost all cases. RpsL43 is paired with a mutation in the ribosomal protein S4, RpsD, while RpsL88 is paired with a mutation in the co-chaperonin GroES. The specific mutation found in RpsD is a known compensatory mutation, which, in *E. coli*, restores an efficient translation to cells which also harbor the K43T mutation in RpsL (Björkman et al. 1999). The specific role of the co-chaperonin GroES (also referred to as HtpA in *Legionella*) in *Legionella* is not fully understood, but chaperonins in *Legionella* have been shown to play very different roles in the different phases of the *Legionella* lifecycle. The chaperonin itself (GroEL/HtpB) appears to be exposed on the surface of the bacterial cell during infection and can trigger phagocytosis in non-phagocytosing cells, while inside cells it can redirect vesicular and organelle trafficking in the host cytoplasm (Garduno et al. 2011). The function of the co-chaperonin GroES is less understood, but it is reasonable to assume that it co-translocates with GroEL. That specific mutation might also be compensatory, e.g. by helping to fold a potentially destabilized mutant RpsL protein.

RpsL, besides its function as a ribosomal protein, may trigger Mϕ death, suggesting an adaptive role in host infection. Through pattern recognition receptors (PRRs), mammalian cells recognize bacterial invaders and trigger cellular apoptosis and inflammatory response (Akira et al. 2006). Many PRRs have known ligands (e.g. flagellin, needle of the type III secretion system), but some are still evasive. Using mouse bone marrow-derived macrophages (BMDMs) as a model, Zhu et al. (2015) suggests that RpsL is responsible for Mϕ death caused by *L. pneumophila*. Strikingly, in that study, only one of the two RpsL mutations found in our experiment (K88R) did not trigger cell death, allowing *Legionella* to replicate. Although we do not know how conserved this interaction is nor whether human cells or amoebae possess a similar mechanism, it is interesting to note that 4 out of 6 of the Mϕ lineages display a fixed RpsL K88R mutation, while it is fixed in 2 *Ac* and 1 Alt lineages (other *Ac* lineages have two subpopulations with each one RpsL mutation). This suggests that, even in human Mϕ, the K88R RpsL mutant might be less prone to trigger cell death. However, competition experiments do not seem to corroborate this observation: the RspL88/GroES genotype provides an important advantage in *Ac* cells but a slight disadvantage in Mϕ (Figure 4).

The RpsL43/RpsD mutation occurred twice in the lineages exposed to Mϕ. We could not test the fitness of that genotype alone since all the isolates we collected had one or more extra mutations. An isolate with an extra IolE mutation showed an increased fitness in *Ac* cells but a decreased one in Mϕ (**Figure 4**), a pattern similar to the RpsL88/GroES genotype. It is counterintuitive that a mutation with a fitness cost (in Mϕ) would be fixed in all populations. A potential explanation is that the *Legionella* populations might have been exposed to streptomycin during the evolution experiment, setting a strong evolutionary pressure. However, streptomycin (and thus the pressure) was absent during the competition experiment. These discordant results might be due to the RpsL mutations being always beneficial in presence of (even traces of) streptomycin, and being beneficial in amoebae but detrimental in Mϕ in the absence of streptomycin.

Supplementary Tables

Supplementary Table 1: Population sequencing and number of generations in the different hosts.

Host	Abbrev.	t1		t2		t3	
		Passages	N generations	Passages	N generations	Passages	N generations
A. castellanii	*Ac*	p10	128 +/- 7	p40	517 +/- 9	p65	798 +/- 9
Alternation	Alt	p10	124 +/- 5	p49	481 +/- 13	p85	799 +/- 13
U937 cells	Mϕ	p15	113 +/- 5	p38	270 +/- 10	p65	444 +/- 40

Supplementary Table 2: Mutation rates (mutations per cell per cell division or generation) measured for *L. pneumophila* Paris, dTomato-, and SYFP2-labeled strains. Mutation rates were measured using streptomycin as the selection antibiotic and then calculated using MSS Maximum Likelihood method. Mutations rates are expressed per cell per generation per billion. CI, Confidence Interval.

Strain	Mutation rate × 10^{-10}	95% CI range × 10^{-10}	
		Upper Bound	Lower Bound
L. pneumophila Paris	6.5	8.5	4.7
dTomato	7.6	9.9	5.5
SYFP	5.5	7.3	3.8

Supplementary Table 3: Clones and mutations involved in competition experiments. Genes and mutations are color-coded.

Strain	Genotype
DA75302	RpsL(K88R)/GroES(A45T)
DA76739	RpsL(K43T)/RpsD(S205Y) + IolE(G241V)
DA76735	RpsL(K88R)/GroES(A45T) + lpp0833(Δ1bp_1092nt)
DA76737	RpsL(K88R)/GroES(A45T) + lpp0833(Δ1bp_1092nt) + LerC_61(Δ13bp 61-73)

DA75291i	RpsL(K43T)/RpsD(S205Y) + LerC_46(C46*)
DA75299i	RpsL(K88R)/GroES(A45T) + lpp0833(Δ1bp_1092nt) + LerC_51(V51G)
LG300i	RpsL(K43T)/RpsD(S205Y) + LerC_13(A13D)

Supplementary Figures

Supplementary Figure 1

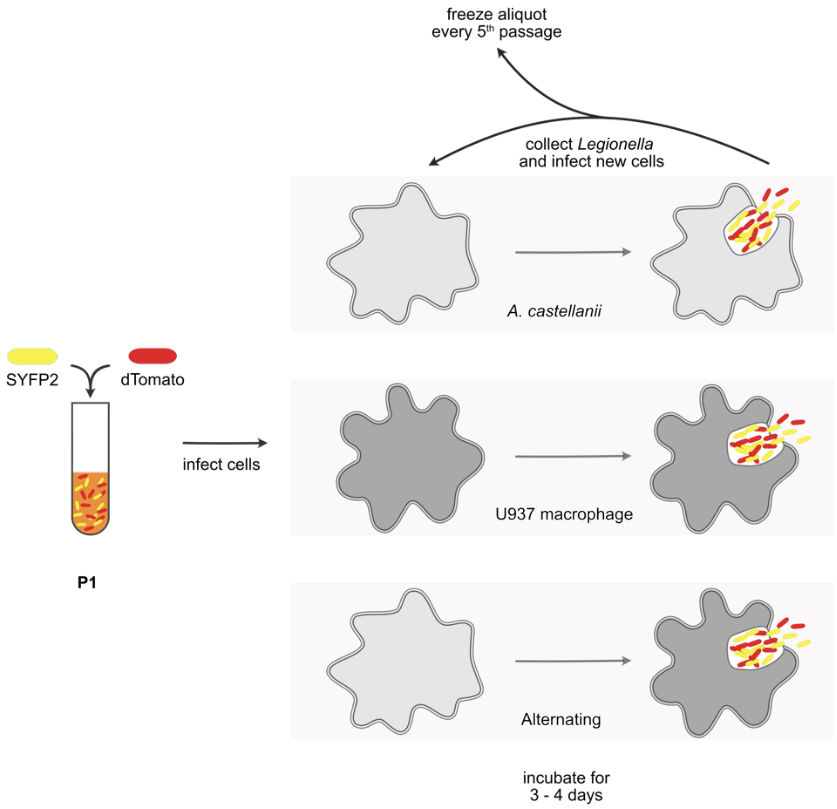

Supplementary Figure 1: Overview of the experimental setup used in this study. A 50/50 mix of SYFP2- and dTomato-tagged *L. pneumophila* Paris was used to start the evolution experiment, with three conditions: passaging the bacteria in *A. castellanii* (*Ac*), in U937 macrophage (Mϕ), and alternating between the two hosts (Alt). For each condition, 6 populations (A-F) were started. Bacterial cells were collected after each passage (3-4 days) and an aliquot was used to reinfect fresh host cells. Aliquots were frozen every 5th passage.

Supplementary Figure 2

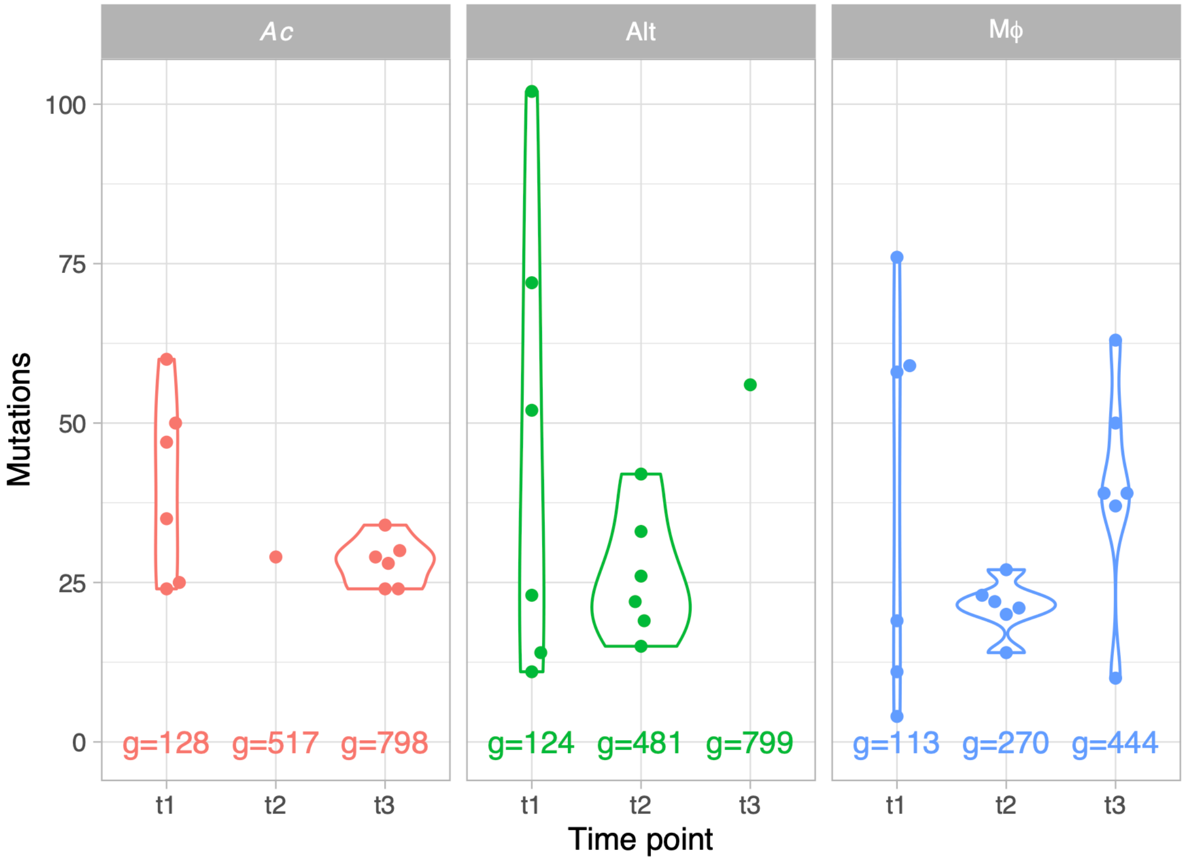

Supplementary Figure 2: Distribution of mutations per host and time point. Each point is a lineage. Only one *Ac* population and was sequenced at t2, and only one Alt population was sequenced at t3. The approximate number of generations for each time point is indicated at the bottom of the plot.

Supplementary Figure 3

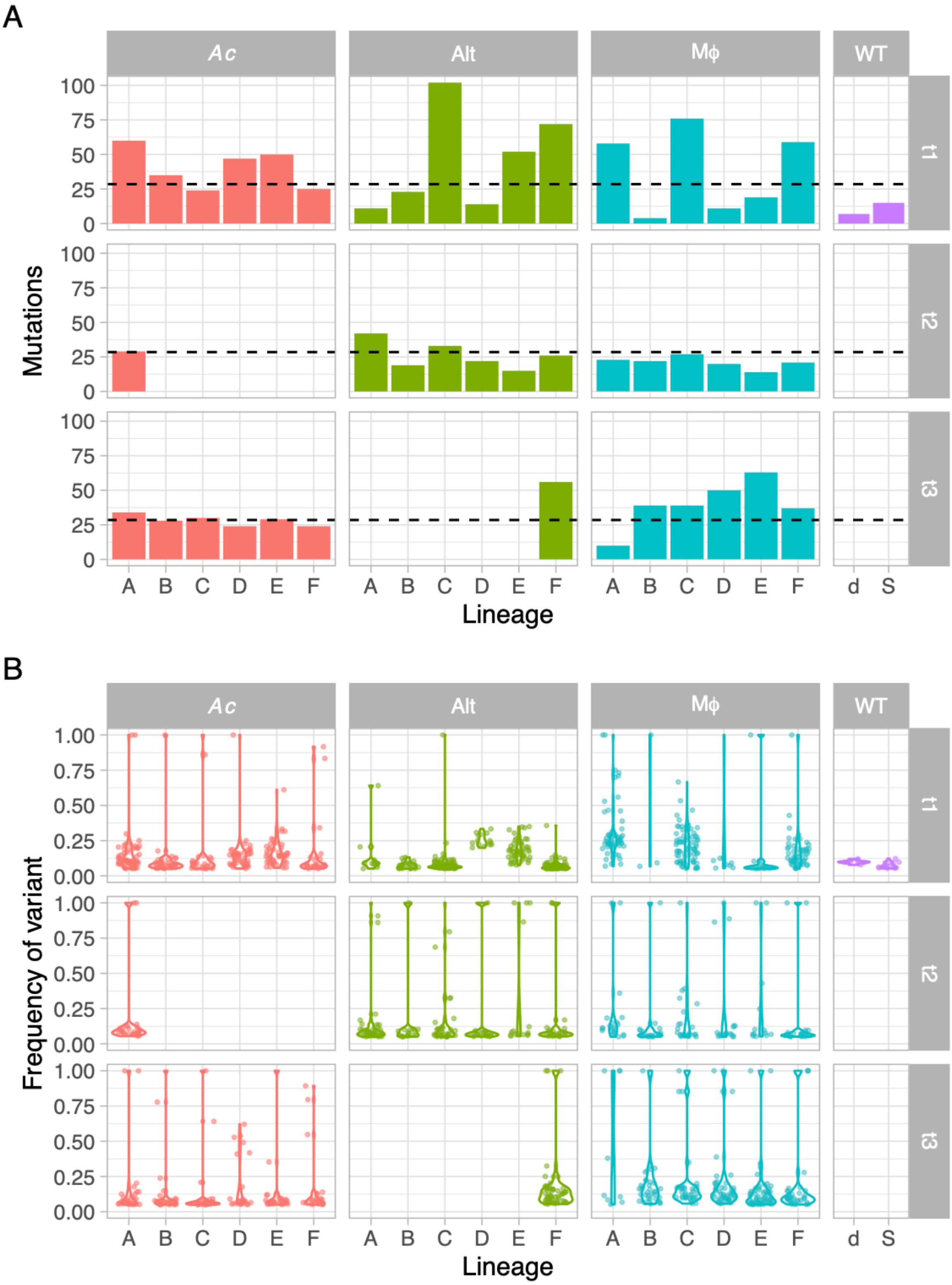

Supplementary Figure 3: Distribution of mutation numbers and frequencies. (A): Number of mutations with frequency > 5% per host (*Ac*, red; Alt, green; Mϕ, teal), lineage (A-F, x-axis) and time point (t1-t3). The median number of mutations per population (32.5) is shown with a dashed black line. The rightmost panels show

mutations found in the control mapping of reads from the two fluorescent ancestors (S, SYFP2; d, dTomato) to the reference genome (WT).

(B): Frequency (y-axis) of mutations per host, lineage and time point, as in A. Each dot represents a mutation, and its position on the y-axis represents its frequency. Violin plots are superimposed on the dots to provide a representation of the distribution.

Supplementary Figure 4

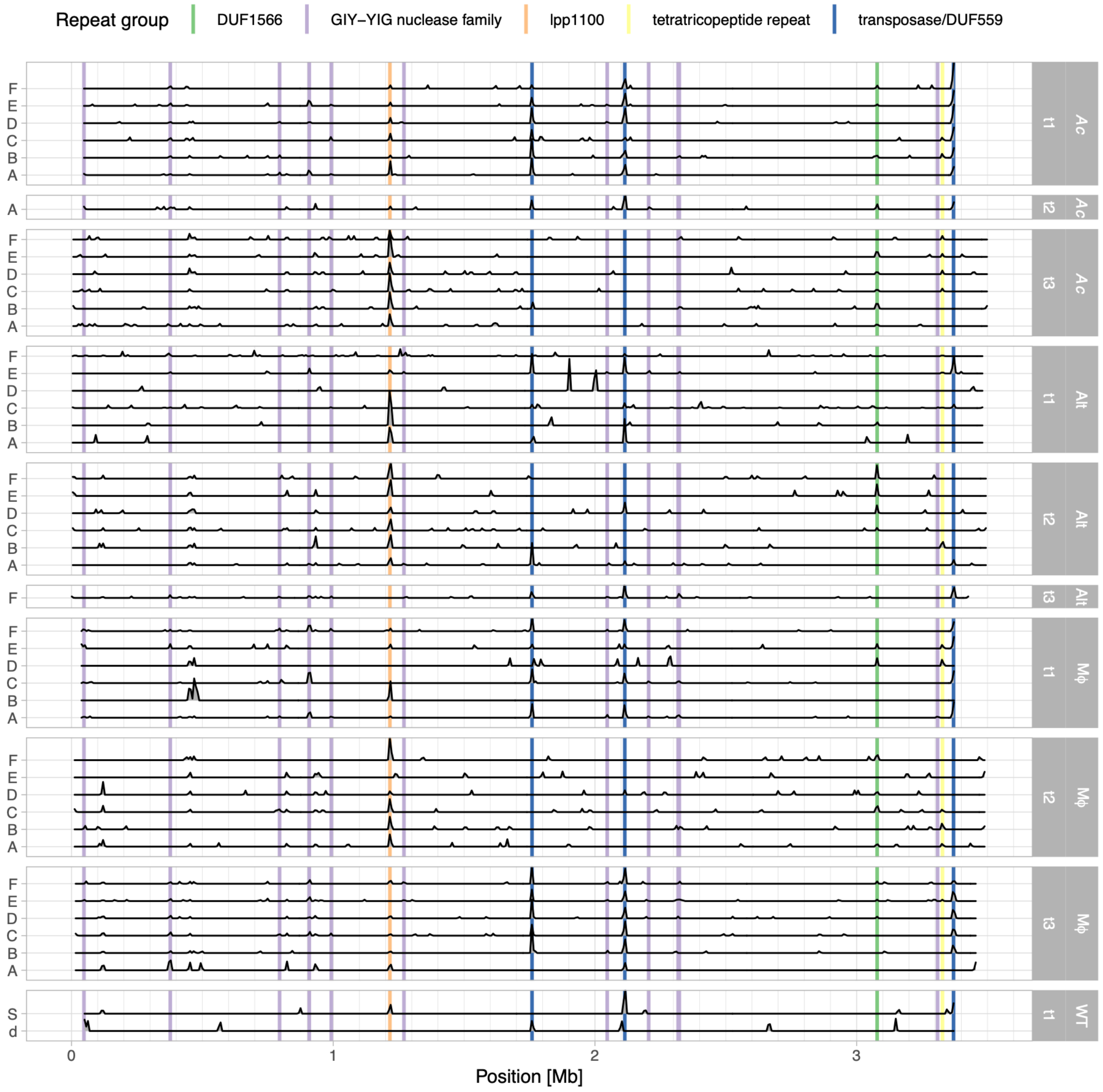

Supplementary Figure 4: Distribution of mutations along the *L. pneumophila* genome. Each line corresponds to a separate population. The host and timepoint is indicated on the right, the lineage on the left. The position of genes containing repeats or being present in different parts of the genome are indicated with lines. The bottom panel (WT) shows the position of artifactual mutations found when mapping the reads from the two fluorescent ancestors (S, SYFP2; d, dTomato) to the reference genome.

Supplementary Figure 5

```
--------------------------------------------------------------------------
No. of Repeats|Total Score|Length  |Diagonal| BW-From|   BW-To|   Level
            19|    1211.97|      35|      35|    1149|    1183|       1
--------------------------------------------------------------------------
  704-  738 (54.25/33.12)    LGALSVEQRVELL.VVQDQHRNTVLHKTLS.RPELLK
  741-  775 (60.83/38.15)    LEGLSLEQRVELL.KVKGSDGNIVLHTVAS.HPKPLK
  778-  812 (66.27/42.31)    LEGLSVEQRVELL.KVKGSDGNTLLHFAAS.NPKSLK
  815-  850 (51.78/31.23)    LEGLPEESRVELLtTLRRKDGASVLDFAAT.HPKSLK
  853-  887 (62.56/39.47)    WESLSEEKRIEVL.EMNDEHGNTVLHWAVS.RPKSLE
  890-  924 (59.21/36.91)    LERLSDEQRFKLL.WMKVQFGNTVLHLAAN.SPETLK
  927-  961 (59.88/37.42)    LEGLPEEKRIEVL.TILNQYGDTVIHRAAH.FPASLK
  964-  998 (67.66/43.37)    WEGLPEEKRIELL.RMKGNYNNTLLHNAAS.HPESLK
 1001- 1035 (58.64/36.48)    LEGLSEQQRAELL.RINGSIGNTVLHCALG.YLESLK
 1038- 1072 (72.67/47.20)    LEGLSEESRFQLL.GMKNQDDNTVLYLAAS.RPESLK
 1075- 1109 (70.30/45.39)    LEDLSVENRVQLL.GMKNQYGNTVLHEALS.QPKSLK
 1112- 1146 (73.90/48.14)    LEGLSEESRFQLL.RMKNQYGDTVLHEALS.QPKSLK
 1149- 1183 (75.50/49.36)    LEGLSEESRVQLL.GMKNQDGYTVLHKALS.LPESLK
 1186- 1220 (75.50/49.36)    LEGLSEESRVQLL.GMKNQDGYTVLHKALS.LPESLK
 1223- 1257 (73.09/47.52)    LEGLSEESRVQLL.GMKDYHGNTLLHEALS.RPKSLK
 1260- 1294 (75.46/49.33)    LEGLSEESRFQLL.GMKNQDGNTVLYLAAS.RPESLK
 1297- 1331 (66.54/42.52)    LEGLSEESRVQLL.GMKDKYGNTALRCAVP.HPASLK
 1334- 1369 (60.00/37.51)    LEGLSEEKRLELL.SAKNQHGDSVLHRALScYPESLG
 1372- 1403 (27.93/13.00)    LEMLCETTQRQVLeDLGCLRGNVFLGK.....PEILE
--------------------------------------------------------------------------

  704-  738 CTTGGCGCATTGTCCGTGGAGCAACGAGTCGAACTGCTT...GTGGTGCAGGACCAACATCGCAACACTGTACTGCACAAGACTTTATCT...CGCCCCGAATTACTGAAA
  741-  775 TTGGAGGGTTTGTCTTTAGAGCAACGAGTCGAACTACTG...AAAGTGAAGGGTAGCGATGGTAATATCGTACTGCACACGGTTGCATCT...CACCCCAAACCCCTGAAA
  778-  812 TTGGAGGGCTTGTCTGTAGAGCAACGAGTCGAACTACTG...AAAGTGAAGGGTAGCGATGGCAATACCCTACTGCATTTCGCTGCATCT...AACCCTAAATCCCTGAAA
  815-  850 TTAGAGGGCTTGCCTGAAGAGAGCCGAGTCGAACTGCTTACTACGTTGCGGAGAAAAGATGGTGCAAGCGTACTGGACTTCGCTGCGACT...CACCCCAAGTCTCTGAAA
  853-  887 TGGGAAAGTTTGTCCGAAGAGAAACGAATCGAAGTGCTG...GAGATGAATGACGAACATGGCAATACTGTCTTGCACTGGGCTGTATCT...AGGCCCAAATCCCTGGAG
  890-  924 TTAGAACGTTTGTCTGACGAGCAACGATTTAAACTCTTG...TGGATGAAGGTTCAATTTGGCAATACTGTACTGCACTTGGCTGCAAAT...AGCCCCGAAACCCTGAAG
  927-  961 TTGGAGGGTTTGCCTGAAGAGAAACGAATCGAAGTGCTG...ACAATCCTGAACCAATATGGCGATACCGTAATACACAGGGCTGCACAT...TTCCCCGCATCCCTGAAA
  964-  998 TGGGAAGGTTTGCCCGAAGAGAAACGAATCGAACTGCTG...AGGATGAAGGGCAATTATAACAATACATTACTGCACAACGCTGCATCT...CATCCCGAATCCCTGAAA
 1001- 1035 TTGGAGGGTTTGTCTGAACAGCAACGAGCCGAACTACTG...AGGATAAATGGTAGCATTGGCAATACTGTACTGCACTGCGCATTAGGT...TATCTGGAATCCCTGAAA
 1038- 1072 TTGGAGGGTTTGTCTGAAGAGAGCCGATTCCAACTGCTG...GGGATGAAGAACCAGGATGACAATACTGTACTGTACCTCGCTGCGTCT...CGACCCGAATCCCTGAAA
 1075- 1109 TTGGAGGACTTGTCTGTAGAGAACCGAGTCCAACTGCTG...GGGATGAAGAACCAATATGGCAATACCGTACTGCATGAGGCTTTATCT...CAACCCAAATCCCTGAAA
 1112- 1146 TTGGAGGGTTTATCTGAAGAGAGCCGATTCCAACTGCTG...AGGATGAAGAATCAATATGGTGATACCGTACTGCATGAGGCTTTATCT...CAACCCAAATCCCTGAAA
 1149- 1183 TTGGAGGGGTTGTCTGAAGAGAGCCGAGTCCAACTGCTG...GGGATGAAGAACCAAGATGGCTATACCGTACTGCATAAGGCTTTATCT...CTACCCGAATCTCTGAAA
 1186- 1220 TTGGAGGGGTTGTCTGAAGAGAGCCGAGTCCAACTGCTG...GGGATGAAGAACCAAGATGGCTATACCGTACTGCATAAGGCTTTATCT...CTACCCGAATCTCTGAAA
 1223- 1257 TTGGAGGGGTTGTCTGAAGAGAGCCGAGTCCAACTACTG...GGGATGAAGGACTATCATGGCAATACTTTACTGCATGAGGCTTTATCT...CGACCAAAATCCCTGAAA
 1260- 1294 TTGGAGGGCTTGTCTGAAGAGAGCCGATTCCAACTGCTG...GGGATGAAGAACCAGGATGGCAATACTGTACTGTACCTCGCTGCGTCT...CGACCCGAATCCCTGAAA
 1297- 1331 TTGGAGGGCTTGTCTGAAGAGAGCCGAGTCCAACTGCTG...GGGATGAAGGACAAATATGGAAATACCGCACTGCGCTGCGCTGTGCCT...CACCCCGCATCCCTGAAA
 1334- 1369 TTGGAGGGTTTGTCTGAAGAGAAACGATTGGAACTGCTT...AGCGCGAAGAACCAGCATGGCGATTCCGTATTGCATAGGGCTTTATCATGTTATCCTGAATCTTTAGGG
 1372- 1403 CTAGAAATGCTGTGTGAGACAACTCAGAGGCAAGTGCTCGAGGATTTGGGATGTTTGCGTGGCAATGTATTCTTAGGTAAGCC...............TGAAATCCTAGAG
------------------------------------------------------------------------------------------------------------------------
```

Supplementary Figure 5: Repeats in ankyrin-repeat containing protein lpp1100. The analysis was performed using RADAR (Heger and Holm 2000). The upper panel shows the location of the repeats (along the protein), the alignment and *Z*-scores, and the amino acid sequence of the repeats. The lower panel shows the same repeats, but in nucleotide space. Only two repeats (1186- 1220 and 1149-1183) are completely identical.

Supplementary Figure 6

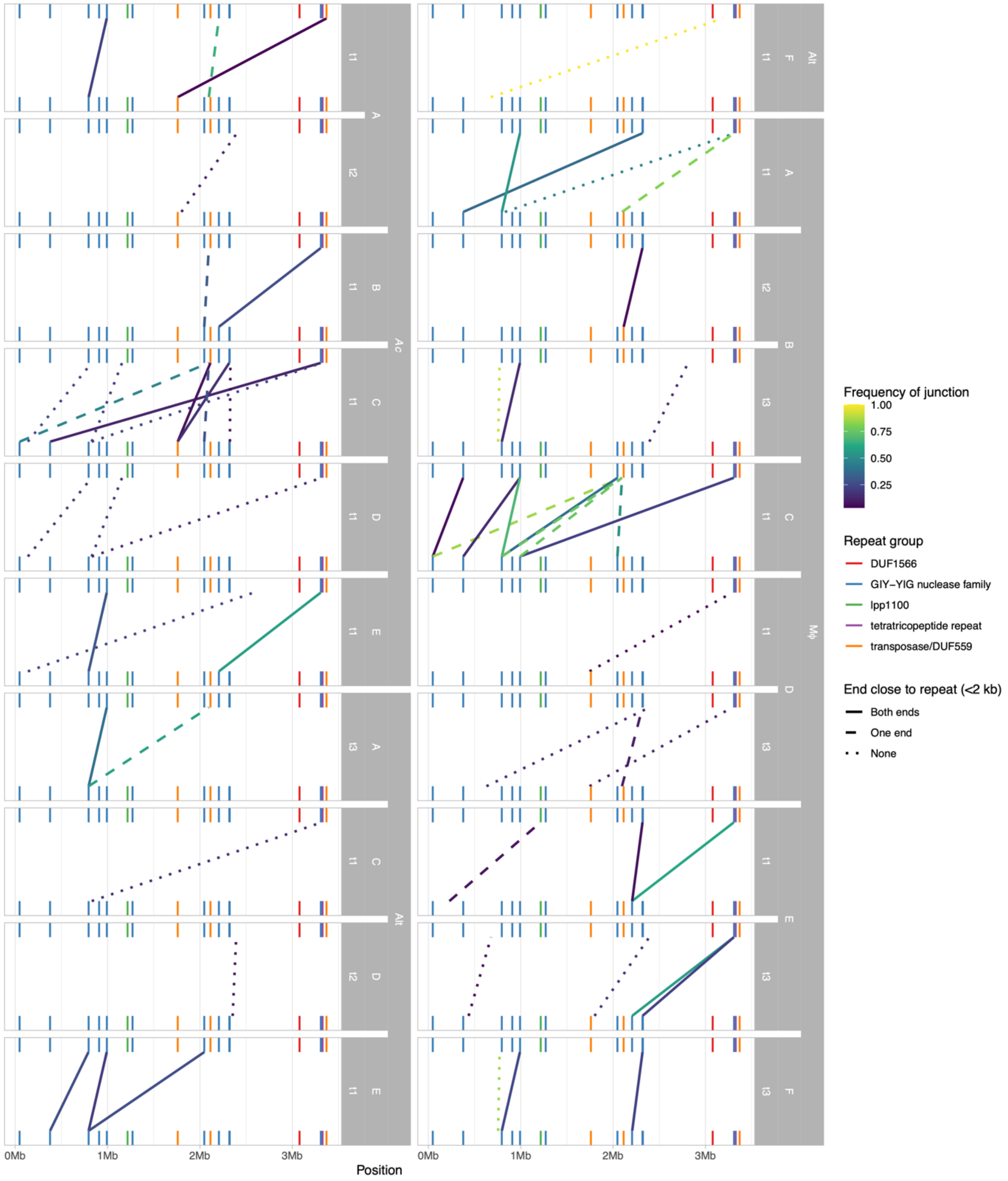

Supplementary Figure 6: Recombinations detected by breseq in 10 of the 38 populations analyzed in this study. The condition (host), lineage and timepoint of the population is indicated to the right of each panel. On each panel, the x-axis shows the position on the genome. Bars at the top and bottom of the panel indicate the position of the recombination hotspots (repeat groups). Different colors indicate different groups. Lines in the middle of the panel indicate potential recombinations, or junctions, as detected by breseq. The position of each end corresponds to where on the chromosome they map. The line is solid, dashed or dotted if both, one or no ends, respectively, map less than 2 kb from a recombination hotspot. The color of

the line reflects the frequency at which the junction was detected in the population. For readability, junctions whose ends map less than 5 kb from each other are not shown on this figure.

Supplementary Figure 7

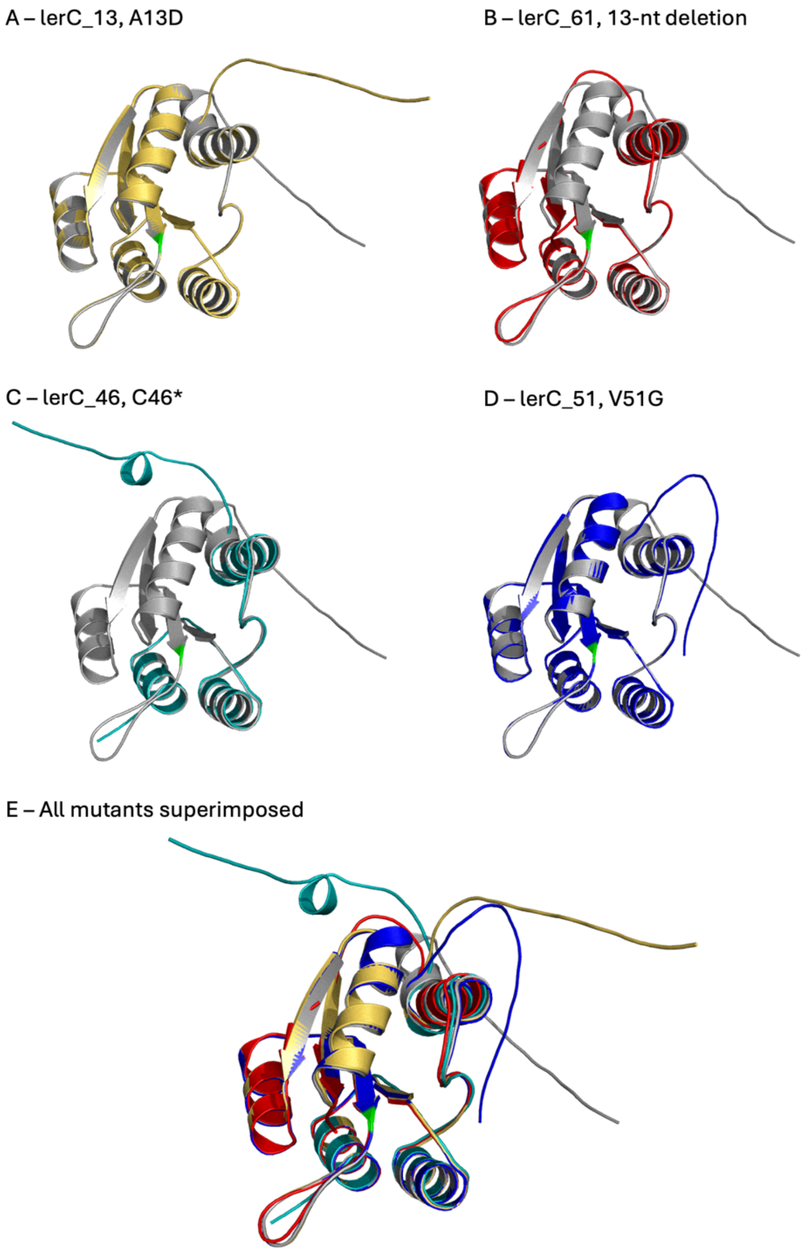

Supplementary Figure 7: Structure of the *lerC* mutants, as predicted by AlphaFold3. The predicted wild-type LerC protein is shown in grey on all panels, lerC_13 in yellow (**A, E**), lerC_61 in red (**B, E**), lerC_46 in teal (**C, E**), and lerC_51 in blue (**D, E**). The location of the conserved aspartic acid (D53) is shown in green.

Supplementary Figure 8

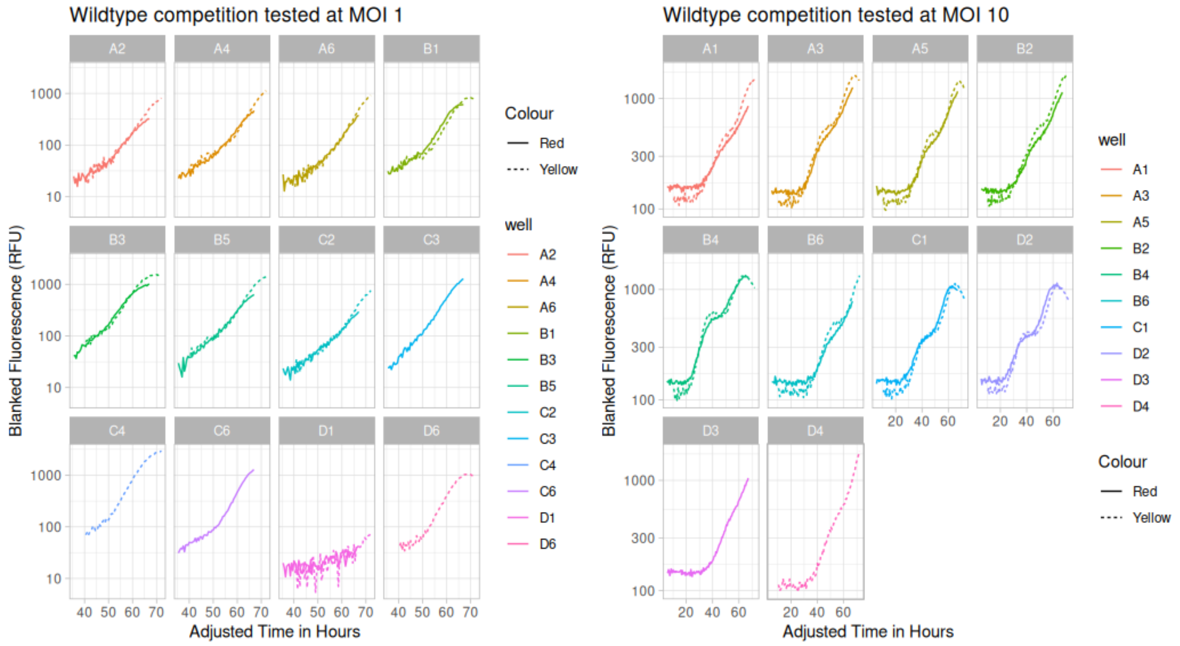

Supplementary Figure 8: Competition experiments between selected isolates. Competitions of *L. pneumophila* strain Paris marked with dTomato (Red) and SYFP2 (Yellow) in an amoeba infection model at MOI 1 and MOI 10.

Supplementary Figure 9

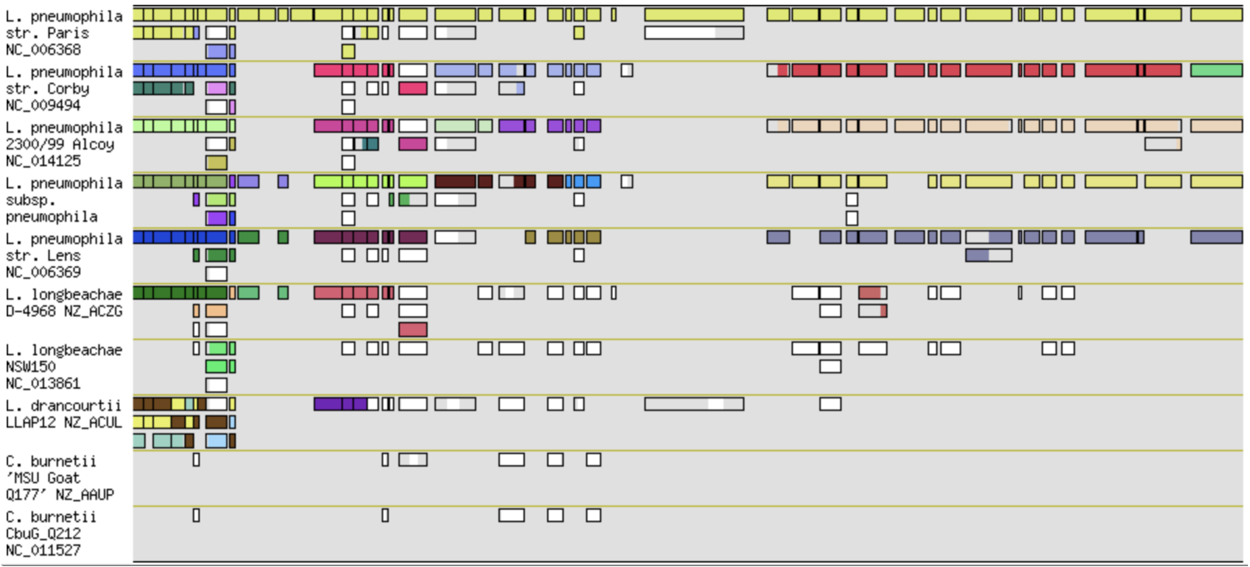

Supplementary Figure 9: Synteny plot of *Legionella* and *Coxiella* species, centered around lpp1100 (LPP_RS05510) in *L. pneumophila* Paris (upper row). The other rows Colors indicate groups of genes colocalized in a specific genome. Figure obtained with MAGE (Vallenet et al. 2006).

Supplementary Figure 10

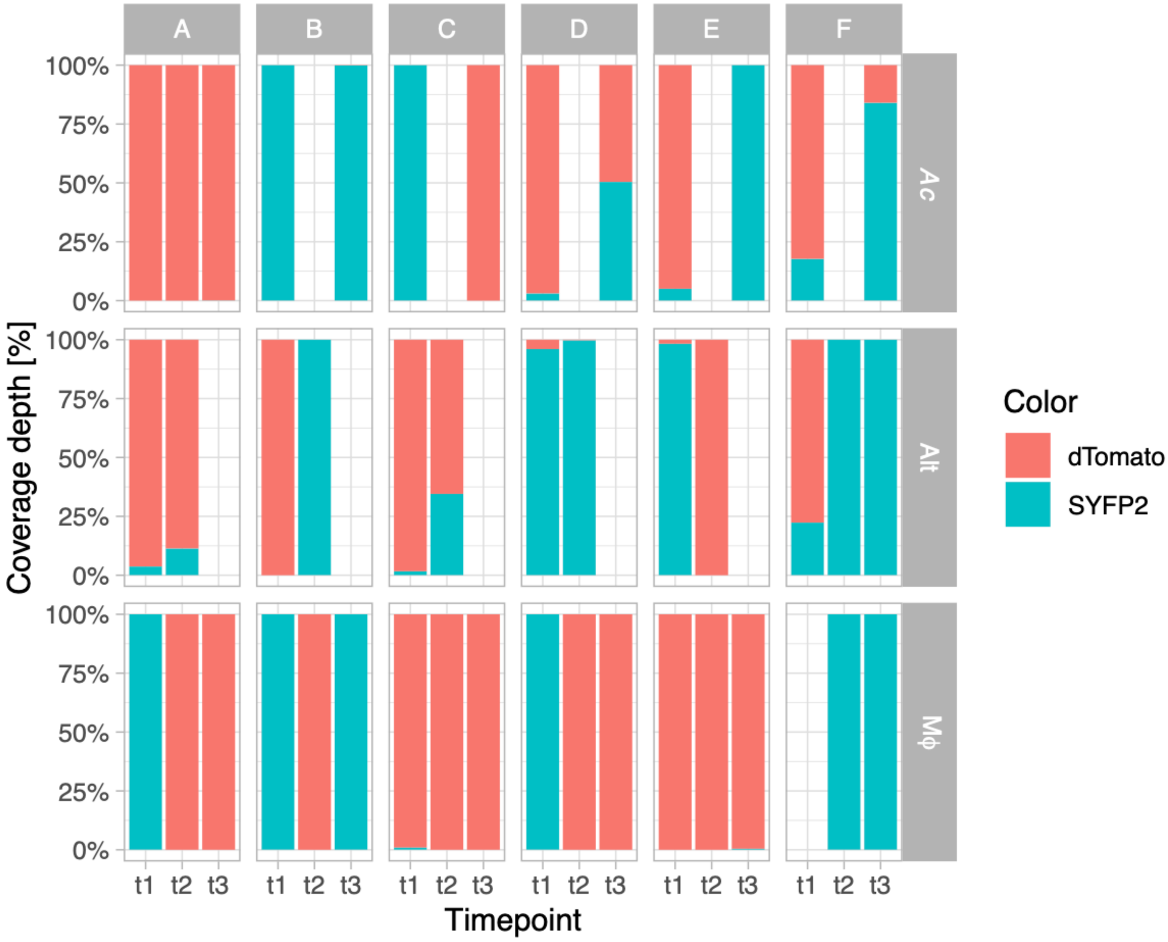

Supplementary Figure 10: Proportion of SYFP2- and dTomato-marked *L. pneumophila* in each lineage at the 3 time points. At t0, each population consisted of an equal mix of each ancestor. The proportion at the other time points was estimated by mapping the reads obtained from population sequencing to the genes encoding the two fluorescent proteins (dTomato, red; SYFP2, green).

Supplementary Figure 11

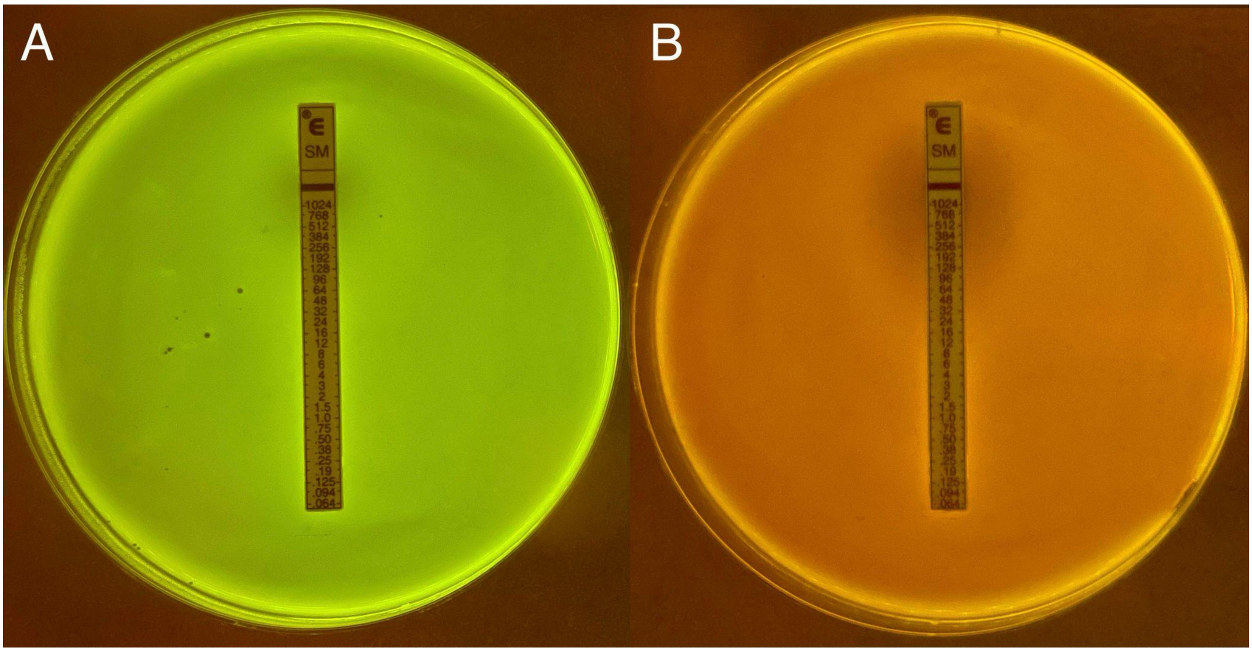

Supplementary Figure 11: Resistance to streptomycin of two *Legionella* clones bearing different mutations in the *rpsL* gene. (A) *L. pneumophila* with the Rpsl43/RpsD genotype, in a SYFP2 background. (B) *L. pneumophila* with the Rpsl88/GroES genotype, in a dTomato background. Although the bacteria grow on the whole plate, including at the maximum streptomycin concentration (1024 µg/ml), a larger halo zone can be observed in B (to ~96 µg/ml) than in A (to ~768 µg/ml).

Supplementary Figure 12

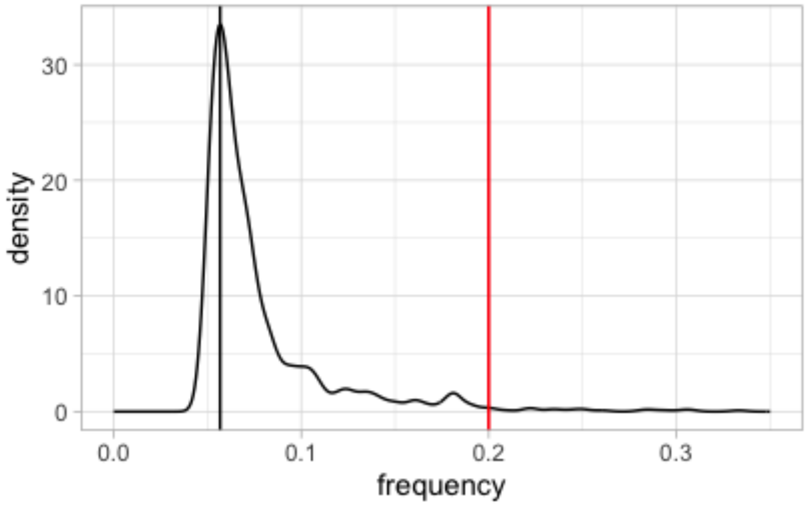

Supplementary Figure 12: Distribution of mutation frequencies in the Alt D population, which counted 991 mutations. The black vertical line marks the maximum of the density curve, at 5.7%, while the red line marks the chosen 20% cut-off, under which mutations were discarded.

Supplementary Figure 13

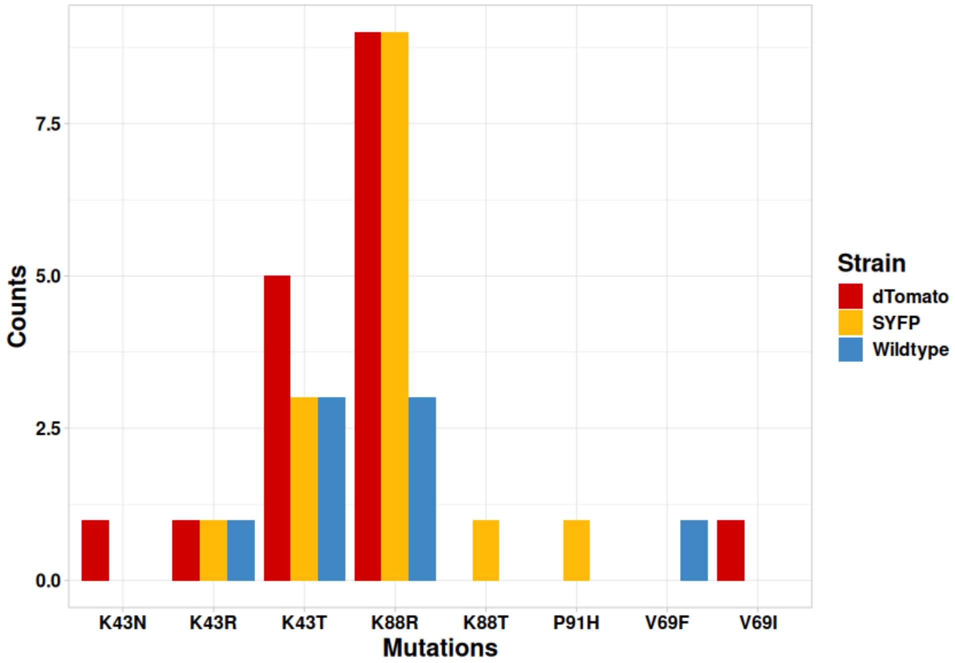

Supplementary Figure 13: Distribution and diversity of non-synonymous mutations in the *rpsL* gene for the three ancestral strains (*L. pneumophila* Paris, SYFP, and dTomato). The x-axis represents the amino acid residues switched through the mutation and their position in the RpsL sequence.

References

Akira S, Uematsu S, Takeuchi O. 2006. Pathogen Recognition and Innate Immunity. *Cell* 124:783–801.

Berger KH, Isberg RR. 1993. Two distinct defects in intracellular growth complemented by a single genetic locus in Legionella pneumophila. *Mol. Microbiol.* 7:7–19.

Björkman J, Samuelsson P, Andersson DI, Hughes D. 1999. Novel ribosomal mutations affecting translational accuracy, antibiotic resistance and virulence of Salmonella typhimurium. *Mol. Microbiol.* 31:53–58.

Funatsu G, Wittmann HG. 1972. Ribosomal proteins: XXXIII. Location of amino-acid replacements in protein S12 isolated from Escherichia coli mutants resistant to streptomycin. *J. Mol. Biol.* 68:547–550.

Garduno RA, Chong A, Nasrallah GK, Allan DS. 2011. The Legionella pneumophila Chaperonin – An Unusual Multifunctional Protein in Unusual Locations. *Front. Microbiol.* 2:122.

Gullberg E, Cao S, Berg OG, Ilbäck C, Sandegren L, Hughes D, Andersson DI. 2011. Selection of Resistant Bacteria at Very Low Antibiotic Concentrations. *PLOS Pathog.* 7:e1002158.

Heger A, Holm L. 2000. Rapid automatic detection and alignment of repeats in protein sequences. *Proteins Struct. Funct. Bioinforma.* 41:224–237.

Hubber A, Kubori T, Coban C, Matsuzawa T, Ogawa M, Kawabata T, Yoshimori T, Nagai H. 2017. Bacterial secretion system skews the fate of Legionella-containing vacuoles towards LC3-associated phagocytosis. *Sci. Rep.* 7:44795.

Nair J, Rouse DA, Bai G-H, Morris SL. 1993. The rpsL gene and streptomycin resistance in single and multiple drug-resistant strains of Mycobacterium tuberculosis. *Mol. Microbiol.* 10:521–527.

Rao C, Benhabib H, Ensminger AW. 2013. Phylogenetic Reconstruction of the Legionella pneumophila Philadelphia-1 Laboratory Strains through Comparative Genomics. *PLOS ONE* 8:e64129.

Timms AR, Steingrimsdottir H, Lehmann AR, Bridges BA. 1992. Mutant sequences in the rpsL gene of Escherichia coli B/r: Mechanistic implications for spontaneous and ultraviolet light mutagenesis. *Mol. Gen. Genet. MGG* 232:89–96.

Vallenet D, Labarre L, Rouy Z, Barbe V, Bocs S, Cruveiller S, Lajus A, Pascal G, Scarpelli C, Medigue C. 2006. MaGe: a microbial genome annotation system supported by synteny results. *Nucleic Acids Res.* 34:53–65.

Zhu W, Tao L, Quick ML, Joyce JA, Qu J-M, Luo Z-Q. 2015. Sensing Cytosolic RpsL by Macrophages Induces Lysosomal Cell Death and Termination of Bacterial Infection. *PLOS Pathog.* 11:e1004704.
